# Technical system of electroencephalography-based brain–computer interface: Advances, applications, and challenges

**DOI:** 10.4103/NRR.NRR-D-25-00217

**Published:** 2025-09-03

**Authors:** Hui Yu, Qiyue Mu, Chong Liu, Shuo Wang, Jinglai Sun

**Affiliations:** 1 Department of Biomedical Engineering, Tianjin University School of Medicine, Tianjin, China; 2 State Key Laboratory of Advanced Medical Materials and Devices, Tianjin University School of Medicine, Tianjin, China; 3 Haihe Laboratory of Brain-Computer Interaction and Human-Machine Integration, Tianjin University School of Medicine, Tianjin, China; 4 Department of Anesthesiology, Tianjin 4^th^ Center Hospital, The Fourth Center Clinical College of Tianjin Medical University, Tianjin, China; 5 School of Electronics and Information Engineering, Tiangong University, Tianjin, China

**Keywords:** clinical trial, deep learning, diagnosis, electroencephalography paradigms, electrode, motor imagery, rehabilitation, sensor, steady-state visually evoked potential, transfer learning

## Abstract

Electroencephalography-based brain–computer interfaces have revolutionized the integration of neural signals with technological systems, offering transformative solutions across neuroscience, biomedical engineering, and clinical practice. This review systematically analyzes advancements in electroencephalography-based brain–computer interface architectures, emphasizing four pillars, namely signal acquisition, paradigm design, decoding algorithms, and diverse applications. The aim is to bridge the gap between technology and application and guide future research. In signal acquisition, noninvasive systems using wet, dry, and semi-dry electrodes are more comfortable and gentler on the skin compared to traditional methods. However, ensuring stable signal quality over long periods of time remains a challenge. Minimally invasive approaches, such as microneedle arrays and endovascular probes, achieve near-invasive signal fidelity without major surgery. Paradigm design explores task-specific neural encoders. Although motor imagery paradigms are widely used in rehabilitation, they require weeks of user training. Steady-state visually evoked potential and P300 speller paradigms enable rapid calibration, but cause visual and cognitive fatigue. Advanced systems currently combine electroencephalography with electromyography or eye-tracking to better handle real-world tasks. Decoding algorithms have advanced through Riemannian geometry for improved noise filtering, deep learning architectures for automated spatiotemporal feature extraction, and transfer learning frameworks to minimize cross-subject calibration. However, challenges remain in managing inconsistent electroencephalography, reducing processing demands, and ensuring compatibility across different electroencephalography devices. Clinical trials reveal a predominant focus on stroke rehabilitation, while emerging frontiers include astronaut neuro-monitoring in space exploration. Challenges include improving signal accuracy, minimizing movement interference, addressing ethical data concerns, and ensuring real-world use. Future advancements focus on biocompatible nanomaterials, adaptive algorithms, and multimodal integration, positioning electroencephalography-based brain–computer interfaces as pivotal tools in next-generation neurotechnology.

## Introduction

**Figure 1:**
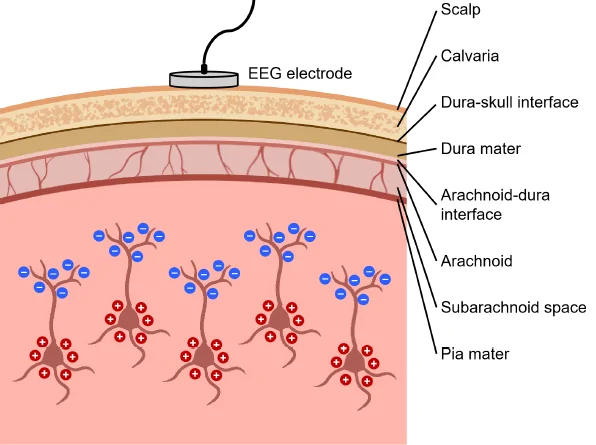
EEG generation mechanism. Scalp EEG records the summed excitatory and inhibitory postsynaptic potentials of thousands of aligned pyramidal neurons near each electrode. Their synchronized activity forms dipolar fields parallel to the orientation of the cells. EEG: Electroencephalography.

Electroencephalography (EEG) captures neuronal electrical activity through EEG electrodes, providing a high-resolution and cost-effective method for analyzing brain states (Altaheri et al., 2023). Scalp EEG measures electrical activity generated by aligned cortical neuron populations near recording electrodes. Most activity originates from pyramidal cells in cortical layers three and five. Recorded signals represent summed excitatory/inhibitory postsynaptic potentials from thousands of these cells. This synchronized activity creates dipolar fields with orientation parallel to pyramidal cell alignment. Negative dipoles perpendicularly oriented toward electrodes produce the the most accurate recordings. Dipole vectors (direction of energy flow) align with pyramidal cell orientation, with maximal detection occurring when negative poles directly face electrodes. The EEG generation mechanism is illustrated in **Figure 1**.

**Figure 2:**
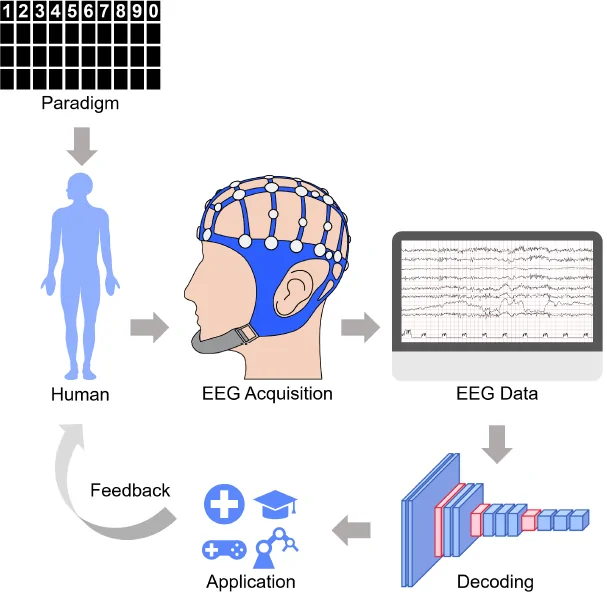
BCI operation principle. Modern BCI systems typically comprise four core components: signal acquisition, paradigm design, signal decoding, and feedback. BCI: Brain–computer interface; EEG: electroencephalography.

This technology forms the foundation of brain–computer interfaces (BCIs), which translate neural signals into external device commands without relying on peripheral nerves (Craik et al., 2019). Modern BCI systems typically comprise four core components (**Figure 2**; Xu et al., 2021): 1) Signal acquisition: Collection of neurophysiological data from subjects; 2) Paradigm design: Modulation of brain activity through task protocols; 3) Signal decoding: Machine learning-driven interpretation of neural patterns; 4) Feedback implementation: Real-time performance visualization for user adaptation.

BCIs demonstrate remarkable versatility, with validated applications spanning rehabilitation (Gao et al., 2023b; Zhu et al., 2023), clinical diagnostics (Sun et al., 2022; Tao et al., 2023), education (Andrews, 2022; Zhou and Baines, 2024), gaming (GomezRomero-Borquez et al., 2024), and intelligent control (Wang et al., 2023a; Zhang et al., 2024f).

While existing research has advanced individual BCI components, a critical gap remains: how to systematically integrate technological advancements across the BCI pipeline to address persistent challenges in signal reliability, decoding efficiency, and real-world adaptability. Most prior reviews focus narrowly on isolated technologies and do not involve clinical trial investigations, leaving unaddressed synergistic opportunities arising from cross-component innovation (Craik et al., 2019; Padfield et al., 2019; Mane et al., 2020; Pan et al., 2022; Liu et al., 2023; Qin et al., 2023; Tibermacine et al., 2024; Zhang et al., 2024e).

This review uniquely offers a holistic framework integrating EEG-BCI technologies and spotlights the transformative role of deep learning through novel architectures. The aim is to bridge the gap between technology and application and guide future research. We systematically examined contemporary EEG-based BCI architectures, analyzed breakthroughs in EEG signal acquisition, paradigms, and decoding algorithms, and discussed emerging applications from medical rehabilitation to aerospace engineering, especially the current clinical trials. Overall, this article offers a broad, systematic overview of EEG-BCI technologies and applications, providing a knowledge framework that updates readers on isolated technical breakthroughs, bridges existing knowledge fragmentation in EEG-based BCI research, and proposes actionable pathways for next-generation system development.

## Search Strategy

Studies cited in this narrative review were searched on the Web of Science Core Collection database using keywords “electroencephalography (EEG),” “brain–computer interface (BCI),” “signal acquisition,” “wet electrode,” “dry electrode,” “semi-dry electrode,” “noninvasive,” “minimally invasive,” “paradigm,” “encoding,” “motor imagery (MI), ”“sensorimotor rhythms (SMR),” “imagined body kinematics (IBK),” “steady-state visually evoked potential (SSVEP),” “visual P300,” “decoding,” “Riemannian geometry,” “deep learning,” “convolutional neural network (CNN),” “recurrent neural network (RNN),” “auto-encoder (AE),” “deep belief network (DBN),” “transformer,” “transfer learning,” “application,” “diagnosis,” “rehabilitation,” “Alzheimer’s disease (AD),” “Parkinson’s disease (PD),” “epilepsy,” “stroke,” “attention deficit and hyperactive disorder (ADHD),” “education,” “gaming,” “autonomous driving,” “exoskeleton,” and “space exploration.” There were no restrictions regarding the year of publication in the search. Diverse combinations of the aforementioned search terms were utilized to conduct a comprehensive identification of relevant scholarly materials. After completing the initial search, titles and abstracts were manually screened and critically reviewed to exclude irrelevant studies. The references included in this review were selected according to the following inclusion criteria: 1) written in the English language; and 2) presenting original empirical evidence or offering theoretical insights of academic value, regardless of publication type or year of publication.

The clinical trial data were sourced from the Chinese Clinical Trial Registry (ChiCTR; https://www.chictr.org.cn/) and ClinicalTrials.gov. The search was conducted up to January 23, 2025.

## Electroencephalography Acquisition

**Figure 3:**
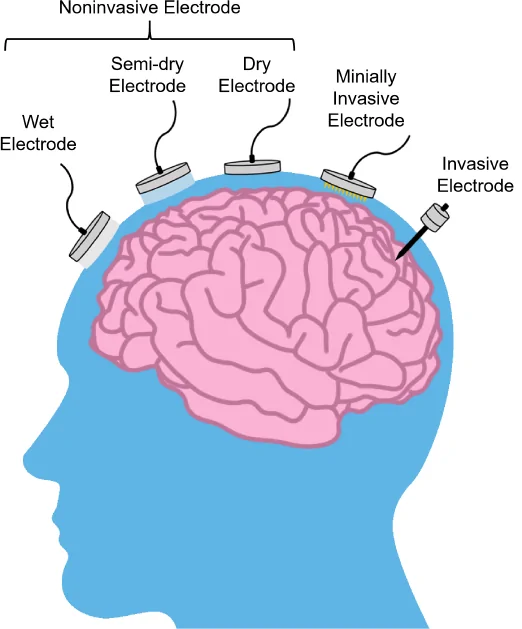
Diagram of invasive, minimally invasive and noninvasive electrodes used in EEG acquisition systems. BCI electrodes are classified into three categories: invasive, semi-invasive, and noninvasive. Noninvasive electrodes can be further divided into wet, dry, and semidry types on the basis of their contact mechanism with the skin. BCI: Brain–computer interface; EEG: electroencephalography.

EEG acquisition, as the cornerstone of the BCI system, uses electrode arrays to capture neuroelectric signals containing cognitive activities. Its signal-to-noise ratio (SNR) and spatial resolution directly determine the feasibility of subsequent processing. EEG acquisition systems are categorized by three criteria, namely implantation method, electrode type, and sensor principle. The implantation approaches are categorized into invasive, minimally invasive, and noninvasive methods (**Figure 3**; He et al., 2020). Classification by electrode type encompasses single electrodes and electrode arrays, while sensor principles encompass photoelectric and electrical modalities.

Noninvasive BCIs have emerged as particularly advantageous among implantation approaches due to their capacity to acquire EEG signals while circumventing procedural risks associated with surgical morbidity or tissue trauma. This safety profile positions them as promising candidates for real-world deployment in everyday settings (Li et al., 2023b). In contrast, invasive BCIs utilizing intracranially implanted electrodes show therapeutic potential for addressing neurological impairments, with clinical translation of these implantable systems anticipated within the forthcoming decade (Bergeron et al., 2023). However, their implementation remains constrained by surgical risks and the inherent complexities of neurosurgical procedures (Reif et al., 2016). Minimally invasive BCIs, which reduce the risk of infection and neural tissue disruption while still requiring surgical implantation, are an emerging and promising category in neural interfaces (Leuthardt et al., 2021).

At the sensor level, fundamental distinctions exist between single electrodes and electrode arrays. Single-channel electrodes function as discrete biosensors capturing localized neuroelectrical activity at defined anatomical coordinates. Conversely, multielectrode arrays employ geometrically organized sensor configurations (typically grid or matrix patterns) to enable spatiotemporal mapping of cortical potentials across broader regions, thereby facilitating topographic analysis of neural activation patterns (Kim et al., 2018).

Modern optical modalities that evaluate regional neurometabolic and neurovascular changes use neurovascular coupling (Iadecola, 2017) mechanisms to indirectly infer neural activity by measuring hemodynamic responses. Three principal techniques have gained prominence, namely intrinsic signal optical imaging, diffuse correlation spectroscopy (Durduran and Yodh, 2014), and functional near-infrared spectroscopy (fNIRS) (Scholkmann et al., 2014). Among these, fNIRS stands out as the most established and rapidly expanding method in BCI applications due to its operational portability and widespread accessibility (Soekadar et al., 2021). Hybrid EEG-fNIRS architectures combine the strengths of the two methodologies, namely high temporal resolution of EEG and superior spatial resolution and noise resistance of fNIRS (Nicolas-Alonso and Gomez-Gil, 2012; Waldert et al., 2012). The integration of EEG and fNIRS stems from the non-interfering nature of bioelectric and near-infrared optical phenomena. Notably, fNIRS-based BCIs predominantly utilize active task paradigms requiring volitional participant engagement (Khan and Hong, 2017), with established protocols employing cognitive challenge tasks as validated biomarkers of cortical activation states (Hong et al., 2015).

From a materials science perspective, EEG electrodes can be divided into rigid and flexible substrate categories. Recent advancements in nanoscience and nanotechnology have particularly revolutionized flexible electrode design, addressing critical limitations of conventional rigid interfaces (Lin et al., 2022; Tang et al., 2023a). These electrodes, in comparison to their rigid counterparts, demonstrate superior epidermal conformability through stress-adaptive deformation mechanics, achieving enhanced skin-contact stability. This innovation in materials translates to measurable improvements in SNR, extended wear tolerance, and reduced dermatological complications—characteristics that collectively advance the feasibility of chronic noninvasive BCI operation (Lin et al., 2023b).

### Noninvasive brain–computer interface

The noninvasive BCI electrodes used to acquire EEG are typically divided into three types (i.e., wet, dry, and semi-dry).

#### Wet electrodes

Wet electrodes, which typically utilize conductive paste and silver/silver chloride (Ag/AgCl)—a material known for its high exchange current density, low polarization, and stable potential—have been refined and developed over the past decade for noninvasive BCIs, particularly in EEG recording applications (Cattarello and Merletti, 2016; Hinrichs et al., 2020). Despite their utility, these electrodes exhibit limitations, including time-consuming application, potential skin irritation, bridging of electrodes due to conductive gel leakage, limited long-term recording capability, and inconvenience in cleaning after examination (Searle and Kirkup, 2000; Pasion et al., 2016). Hydrogel electrolytes have emerged as potential alternatives to commercial wet electrodes, yet their complex implementation requirements and prolonged preparation times restrict their suitability for portable or daily use devices (Pedrosa et al., 2017; Fiedler et al., 2018). Nevertheless, wet electrodes remain indispensable in current BCI research and clinical applications.

#### Dry electrodes

Dry electrodes, fabricated from solid conductive materials such as metals, conductive polymers, and textiles, acquire physiological signals through direct skin contact without preparation, offering rapid deployment and suitability for portable and wearable devices (Di Flumeri et al., 2019). Noninvasive dry electrodes can be classified as contact electrodes and non-contact electrodes (Ferreira and Pimenta, 2024).

**Figure 4:**
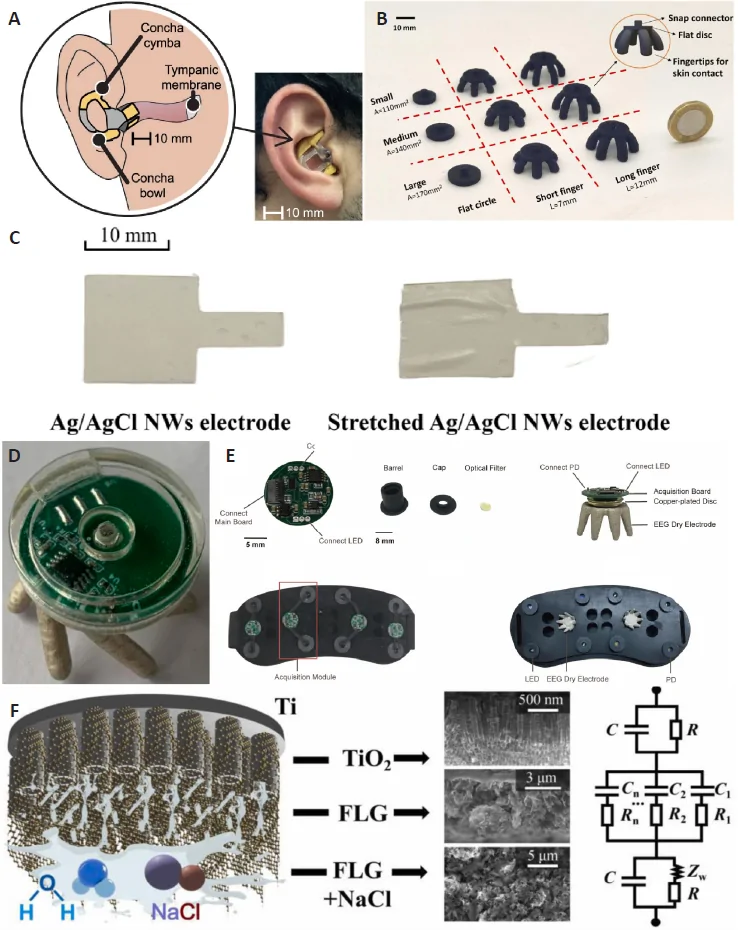
Examples of dry electrodes for EEG-BCI applications. (A) Schematic diagram of the in-ear, dry-electrode earpieces used to monitor drowsiness. Reprinted from Kaveh et al. (2024). (B) Schematic diagram of 3D-printed, flexible, conductive EEG electrodes. Reprinted from Xing and Casson (2023). (C) Photograph of the Ag/AgCl nanowire electrode before and after tensile deformation. Reprinted from Wang et al. (2024d). (D) Photo of an active claw-shaped dry electrode for EEG measurements in hairy areas. Reprinted from Wang et al. (2024g). (E) Photo of a hybrid EEG-fNIRS wearable patch for concurrent brain activity monitoring. Reprinted from Li et al. (2024a). (F) Schematic diagram of the few-layer graphene/TiO_2_ EEG electrode. Reprinted from Li et al. (2023b). Copyright 2023, Elsevier B.V. 3D: Three-dimensional; BCI: brain‒computer interface; CMFF: common-mode feedforward; DCU: digital control unit; EEG: electroencephalography; FLG: few-layer graphene; fNIRS: functional near-infrared spectroscopy; LED: light emitting diode; LOD: leadoff detection; NW: nanowire; OSC: oscillator; PD: photodiode; SCL: serial clock; SDA: serial data.

Dry-contact electrodes are directly placed on the skin, prioritizing user comfort and long-term monitoring feasibility. Recently, in-ear dry-electrode earpieces (**Figure 4A**) were presented for drowsiness detection using compact hardware and machine learning algorithms, achieving comparable classification accuracy (93.2% and 93.3% on existing and unseen users, respectively) to that observed with existing state-of-the-art wet electrode in-ear and scalp systems (Kaveh et al., 2024). This innovation highlights the potential for scalable brain activity monitoring.

Compared with conventional rigid electrodes, flexible dry electrodes have emerged as promising alternatives, addressing limitations such as skin irritation and operational complexity across clinical, research, and everyday applications (Wang et al., 2023b). Three-dimensional (3D)-printed, flexible, conductive EEG electrodes (**Figure 4B**), which are gel- and coating-free, customizable, and more cost-effective than traditional counterparts, have been proposed (Xing and Casson, 2023). Despite their higher impedance than Ag/AgCl electrodes, their cost efficiency and adaptability make them viable for personalized applications. A stretchable Ag/AgCl nanowire dry electrode (**Figure 4C**) was proposed for high-quality multimodal bioelectronic sensing (Zhang et al., 2024e). The electrode, constructed with a flexible network of Ag/AgCl nanowires embedded in polydimethylsiloxane, exhibited a higher SNR than did commercial wet electrodes in electrocardiography (ECG), EMG, and EEG tests under 50% tensile strain. The design ensures enhanced comfort, high sensitivity, and convenience for curved surface biosignal monitoring in clinical contexts. An active claw-shaped dry electrode (**Figure 4D**) was proposed for EEG measurements in hairy areas (Wang et al., 2024g). By incorporating adaptive impedance-matching circuitry, the system achieved low contact impedance (18.62 kΩ at 1 Hz in the hairless region and 122.15 kΩ at 1 Hz in the hair-covered region) and suppressed power-line interference, yielding EEG signals comparable to wet electrodes in quality. A hybrid EEG-fNIRS wearable patch (**Figure 4E**) was proposed for concurrent monitoring of brain activity (Li et al., 2024a). It features reduced spatial interference (input noise to 0.9 µV_rms_, amplitude distortion to less than 2%, and frequency distortion to less than 1%) and a high light-emitting diode switching frequency (above 100 Hz) for crosstalk suppression. This integration enables synchronized acquisition of spatiotemporally coupled brain activity signals.

Noncontact dry electrodes (also called capacitive electrodes) operate without direct skin interaction; instead, they are coupled through dielectric materials such as hair or clothing (Liu et al., 2023). A noncontact EEG electrode based on a few-layer graphene/titanium dioxide (TiO_2_) nanotube nanoarchitecture (**Figure 4F**) was presented for application in robot arm control (Li et al., 2023b). Using a saturated aqueous NaCl solution or sweat as the electronic medium, the electrode achieved a high scalp-contact resistance of 13.4 kΩ, a high SNR of 76.8 dB, and excellent stability over nearly 2 hours of continuous use and a month of long-term use.

#### Semi-dry electrodes

Semi-dry electrodes have emerged as a hybrid solution bridging conventional wet and dry electrodes, combining their advantages while addressing inherent limitations (Li et al., 2020). These electrodes employ an integrated liquid reservoir that continuously supplies electrolyte solution to the scalp, thereby reducing impedance without requiring pre-applied gels or saline-soaked materials (Peng et al., 2016).

**Figure 5:**
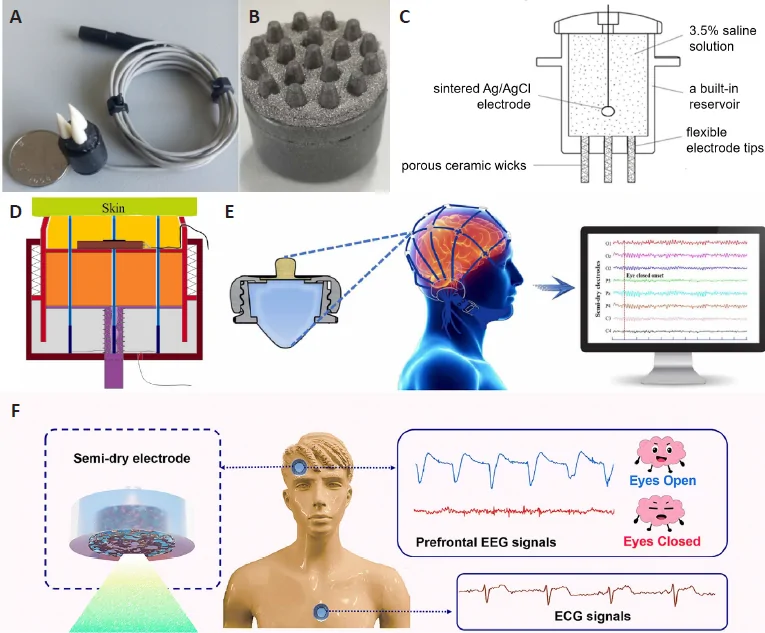
Examples of semidry electrodes for EEG-BCI applications. (A) Photo of a Chinese brush-pen-tip-like microseepage electrode. Reprinted from Xing et al. (2018). Copyright 2017, Elsevier B.V. (B) Photo of a flexible multilayer semidry electrode for long-term scalp EEG measurements. Reprinted from Hua et al. (2019). (C) Schematic diagram of a polymer wick-based semidry electrode. Reprinted from Wang et al. (2016). Copyright 2016, IOP Publishing. (D) Schematic diagram of a novel semidry electrode designed for driving fatigue detection. Reprinted from Wang et al. (2024b). Copyright 2024, Elsevier B.V. (E) Schematic diagram of PVA/PAM DNH-based semidry electrodes for real-world BCIs. Reprinted from Li et al. (2023a). Copyright 2023, IOP Publishing. (F) Schematic diagram of a smart electrolyte-replenishing semidry electrode. Reprinted from Cai et al. (2024a). Copyright 2024, The Royal Society of Chemistry. BCI: Brain‒computer interface; DNH: double-network hydrogel; ECG: electrocardiogram; EEG: electroencephalography; PVA/PAM: polyvinyl alcohol/polyacrylamide.

First-generation semi-dry EEG electrodes facilitate the release of electrolytes through pressure-triggered mechanisms. These electrodes typically deliver a controlled quantity of electrolyte fluid from an integrated reservoir to the scalp via materials that are permeable to electrolytes. In the past 5–6 years, building upon the initial concept (Mota et al., 2013), advanced semi-dry electrodes have been developed for EEG acquisition, which is based on micro-seepage sponge architectures (Xing et al., 2018), flexible multi-layer structures (Hua et al., 2019), and silver-plated electrode cores (Wang et al., 2024a). A Chinese brush-pen-tip-like micro-seepage electrode (**Figure 5A**) with flexible and elastic tips was presented for wearable EEG acquisition, merging dry electrode convenience with wet electrode-level impedance (Xing et al., 2018). A flexible multi-layer semi-dry electrode (**Figure 5B**) was presented for long-term scalp EEG measurements, particularly in hairy areas, offering a promising solution for daily wearable EEG monitoring (Hua et al., 2019). Despite these improvements, first-generation designs suffer from uneven pressure distribution causing premature electrolyte leakage, impedance fluctuations, and challenges in balancing comfort with signal fidelity during prolonged use (Li et al., 2020).

Second-generation semi-dry EEG electrodes employ capillary-driven porous materials for sustained, regulated electrolyte release, eliminating manual reservoir compression (Wang et al., 2016). For example, a novel porous ceramic-based semi-dry electrode (**Figure 5C**) has been introduced (Pedrosa et al., 2018). The core advancement in this semi-dry electrode is its tip design that enables controlled electrolyte permeation through capillary action, creating a self-maintaining ionic conducting path for detecting neural signals. Recently, a novel semi-dry electrode (**Figure 5D**) was proposed for driving fatigue detection, achieving convenient replenishment of conductive liquid, early warning regarding insufficient conductive liquid, longer mechanical service life (6300 times of compression), and more comfortable wear compared to traditional wet and dry electrodes under the premise of low contact impedance (60 kΩ·cm^2^ at 1 Hz and 5 kΩ·cm^2^ at 10 kHz) (Wang et al., 2024b). In summary, the second-generation semi-dry EEG electrodes are capable of delivering conductive fluids in a controlled and sustained manner, eliminating the need for manual pressure application. Nevertheless, there is scope for enhancing user experience, as periodic replenishment of the electrolyte fluid remains necessary.

Hydrogels, characterized by their flexible three-dimensional (3D) polymer chain networks, possess the ability to retain moisture, thereby enhancing the hydration of both hair and scalp, which in turn ameliorates the comfort of wearing such devices (Li et al., 2021; Shen et al., 2021; Pei et al., 2022). Therefore, hydrogel-enhanced semi-dry electrodes represent the next evolutionary stage. A polyvinyl alcohol/polyacrylamide double-network hydrogel electrode (**Figure 5E**) was presented for EEG recording in BCIs, employing cyclic freeze-thaw fabrication to deliver saline for stable impedance (Li et al., 2023a). Validated in a study involving 16 participants, this system achieved a low contact impedance (18 ± 8.9 kΩ at 10 Hz), a small offset potential (0.46 mV), and negligible potential drift (1.5 ± 0.4 µV/min). A thermo-responsive hydrogel semi-dry EEG electrode (**Figure 5F**) was proposed, featuring an autonomous electrolyte-replenishment system activated by body heat (Cai et al., 2024a). The electrode integrates a conductive Ag nanoparticle-loaded sponge/hydrogel heterogeneous interpenetrating network sensing layer, shielded by polydimethylsiloxane and interfaced via Ag/AgCl. This architecture achieved scalp-electrode impedances of 8–18 kΩ without external pressure, enabling prolonged EEG/electromyography (EEG/EMG) recording with 97.9% spectral correlation to wet electrodes—a critical milestone for wearable electrophysiological devices.

### Invasive and minimally invasive brain–computer interface

Invasive BCIs involve surgical implantation of intracortical electrodes to achieve high-fidelity neural signal acquisition, albeit with elevated procedural risks (Reif et al., 2016). In recent years, research on invasive EEG-based BCI has been mostly focused on the columnar invasive EEG electrodes—needle-like structures enabling precise neural activity recording with enhanced spatiotemporal resolution for detailed network analysis (Qin et al., 2023).

**Figure 6:**
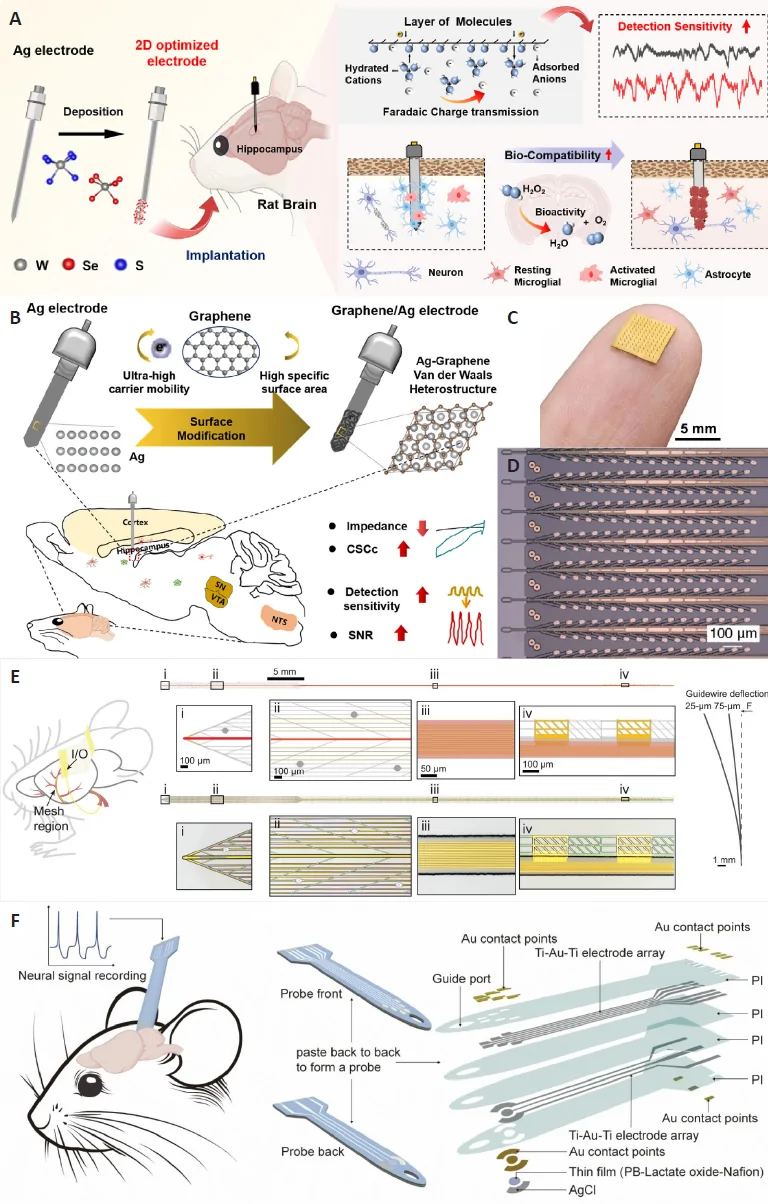
Examples of invasive and minimally invasive BCIs. (A) Schematic diagram of a 2D optimized electrode. Reprinted from Liu et al. (2024b). Copyright 2024, American Chemical Society. (B) Schematic diagram of a neural electrode made from a graphene/Ag van der Waals heterostructure. Reprinted from Liu et al. (2022). Copyright 2022, American Chemical Society. (C) Schematic diagram of a novel flexible MN array electrode that can provide excellent electrode–skin contact and long-term wearability. Reprinted from Li et al. (2022a). (D) Photo of an integrated brain‒machine interface platform. Reprinted from Musk and Neuralink (2019). (E) Schematic diagram of endovascular implantation and overview of an ultraflexible neural probe. Reprinted from Zhang et al. (2023). (F) Schematic diagram of a flexible multimodal integrated neural probe. Reprinted from Zhang et al. (2024b). Copyright 2024, The Royal Society of Chemistry. 2D: Two-dimensional; BCI: brain‒computer interface; CSCc: cathode charge‒storage capacity; EEG: electroencephalography; NTS: nucleus of the solitary tract; SN: substantia nigra; SNR: signal‒to‒noise ratio; VTA: ventral tegmental area.

Two-dimensional (2D) quantum material-modified electrodes (**Figure 6A**) based on tungsten diselenide (WSe2) and tungsten disulfide (WS2) quantum dots were proposed, achieving simultaneous improvements in neural signal fidelity and biocompatibility (Liu et al., 2024b). These interfaces exhibit reduced impedance (210 MΩ·μm^2^ at 1 kHz), increased cathodic charge storage capacity (5.6 mC/cm^2^), and sustained biocompatibility over 4-week implants, positioning them as transformative tools for high-resolution neural interfacing in medical diagnostics and neuroscience applications. A neural electrode produced from graphene/Ag van der Waals heterostructure electrodes (**Figure 6B**) was presented, which enhances neural signal sensitivity and SNR through optimized interfacial electron transport. This electrode is characterized by 6.3× lower impedance (161.4 ± 13.4 MΩ·μm^2^ at 1 kHz) and 48.4× higher cathodic charge storage capacity (24.2 ± 1.9 mC/cm^2^) than commercial Ag electrodes (Liu et al., 2022). Density functional theory calculations confirm faster electron transfer at the interface, leading to enhanced performance *in vivo*.

Minimally invasive BCIs, requiring surgical implantation while mitigating the risk of infection and neural tissue disruption, have gained prominence (Reif et al., 2016; Leuthardt et al., 2021). Current research emphasizes the use of microelectrode arrays—miniaturized grid-based devices for biopotential recording or neuromodulation (Kim et al., 2018).

A polyimide-based flexible microneedle array electrode (**Figure 6C**) was presented. The normalized electrode–skin interface impedance was 0.98 kΩ·cm^2^ at 1 kHz and 1.50 kΩ·cm^2^ at 10 Hz, approximately 1/250 of that recorded with clinical standard electrodes, applied to clinical long-term continuous monitoring for polysomnography such as EEG and ECG (Li et al., 2022a). Although the microneedle electrodes offer stable contact and good signal quality compared to wet electrodes, they may lead to infection or inflammation and are associated with a risk of needle breakage in the body (Ren et al., 2020). The use of new materials or preparation methods does not completely prevent these issues. In 2019, Neuralink was presented, an integrated brain-machine interface platform with thousands of channels, advancing neural tech (Musk and Neuralink, 2019). It uses small and flexible electrode “threads,” each with up to 3072 electrodes across 96 threads, for high-resolution neural recording (**Figure 6D**). A neurosurgical robot has been developed to insert these threads with micron accuracy, avoiding vasculature and targeting brain areas at six threads per minute. With a 70% spiking yield in chronic implants, Neuralink’s system offers high density and scalability for clinical use. It is a cutting-edge research tool and prototype for future human implants, potentially restoring senses and motor functions, as well as treating neurological disorders.

Endovascular signal, a minimally invasive subclass, enables intracranial access via blood vessels (Sefcik et al., 2016). Endovascular methods are less invasive than open surgery, leading to more rapid recovery and lower risk of infection. Devices such as the stent-electrode recording array (Stentrode), which fuses with blood vessel walls, provide stable signals, unlike invasive cortical electrodes that might lose the signal due to gliosis (He et al., 2024). Ultraflexible neural probes (**Figure 6E**) implantable into small blood vessels (< 100 μm) without surgery were presented (Zhang et al., 2023). This approach led to successful *in vivo* recording of brain signals and long-term testing, proving the minimal invasiveness of the probe and its compatibility with the body. A multimodal integrated flexible neural probe (**Figure 6F**) integrating six-channel EEG and lactate biosensors was also proposed (Zhang et al., 2024b). The EEG electrodes had an average impedance of 2.57 kΩ at 1 kHz and remained stable after being immersed in normal saline for 3 months. The lactic acid sensor showed a sensitivity of 52.8 nA/mM. These implanted devices did not exhibit significant perturbations to cerebral microvasculature integrity and neurological function, or the structural integrity of the blood-brain barrier. Their compact design enabled the devices to record local field potentials via the scaffold electrode recording arrays and to capture single-neuron activity. The minimally invasive capability to record from individual neurons is of critical importance for the investigation of deep brain structures.

### Conclusion and prospects

**Additional Table 1** summarizes the advantages and disadvantages of noninvasive, invasive, and minimally invasive EEG electrodes.

**Additional Table 1:** The advantages and disadvantages of noninvasive, invasive and minimally invasive EEG electrodes

Type	Advantage	Disadvantage
Noninvasive	• No surgery needed; high safety for daily use • Portability and wearability (e.g., dry electrodes) • Avoids tissue trauma and infection risks	• Signal quality affected by skin-electrode impedance (e.g., dry electrodes) • Wet electrodes require conductive gel, risking skin irritation or leakage • Lower spatiotemporal resolution
Invasive	• High spatiotemporal resolution for direct neural recording • Ideal for precise neuromodulation and disease research (e.g., epilepsy localization)	• Requires craniotomy with risks of infection, inflammation, and neural damage • Long-term implants may cause gliosis or electrode failure • Technically complex and costly
Minimally	• Reduced surgical trauma and infection risks (e.g., endovascular implants)	• Partial surgical intervention still required (e.g., vascular access)
invasive	• Near-invasive signal quality • Suitable for long-term monitoring (e.g., flexible microneedle arrays)	• Microneedles may break and remain in the body • High development costs and immature technology

EEG: Electroencephalography.

Wet electrodes, while refined for noninvasive BCIs (e.g., EEG recordings), remain limited by practical constraints including cumbersome application processes, risk of skin irritation, conductive gel leakage, restricted long-term stability, and post-use cleaning challenges. Hydrogel electrolytes have been proposed to address these issues; however, their complex preparation protocols and operational requirements hinder integration into portable or daily-use systems. Despite these limitations, wet electrodes retain critical utility in BCI research and clinical applications.

The development of dry electrodes has overcome the limitations of wet electrodes through gel-free operation, rapid deployment, and enhanced wearability (Niu et al., 2021). However, elevated skin-electrode impedance arising from insufficient electrolyte interface compromises signal fidelity (Guger et al., 2012; Fiedler et al., 2022). Active dry electrodes with high-input impedance preamplifiers mitigate this limitation by impedance conversion, thereby decoupling signal quality from contact resistance. Nevertheless, these systems are characterized by limitations, including cost, bulkiness, motion artifact susceptibility (Shad et al., 2020), and prolonged-wear discomfort due to mechanical pressure (Lin et al., 2023b).

Semi-dry electrodes strategically balance wet and dry electrode paradigms by employing minimal electrolyte solutions to maintain stable scalp interfaces without conductive gels (Li et al., 2020). While second-generation designs require periodic manual electrolyte replenishment, next-generation iterations leverage flexible materials for autonomous hydration. Current limitations include insufficient characterization of polarization dynamics, subjective comfort assessments, and unmet needs for integrated signal-enhancing circuitry. Despite promising hybrid functionality, advancements in wireless operation, real-time processing, and machine learning integration remain essential for practical implementation.

There are also challenges in the utilization of semi-dry electrodes. The second-generation semi-dry electrodes require regular electrolyte replenishment by users. The next-generation semi-dry electrodes have automatic charging and discharging capabilities, highly dependent on smart materials (Li et al., 2020). Assessments of non-polarization performance and signal quality are inadequate, and the evaluation of user comfort lacks objective quantification. The integration of specialized electronic circuits to improve signal quality is also an unmet need. Although semi-dry electrodes hold promise in multiple domains, further advancements are necessary for their practical application, especially concerning wireless recording, machine learning, and real-time processing technologies.

Invasive and minimally invasive EEG electrodes (e.g., microelectrode arrays, columnar arrays) enable precise neural circuit interrogation through direct tissue interfacing, proving indispensable for epilepsy localization (Sun et al., 2022) and neurological disorder research (Sobczak et al., 2021). Their deployment necessitates sophisticated microfabrication for durability and micron-scale surgical precision to prevent parenchymal damage. Though technically demanding, these electrodes provide unique spatiotemporal resolution for decoding neural network dynamics (Musk and Neuralink, 2019; Li et al., 2022a; Liu et al., 2022, 2024b).

Though time-consuming, EEG-BCI calibration significantly influences the subsequent accuracy of the entire measurement. Consequently, various approaches aimed at streamlining or completely eliminating the calibration process while maintaining BCI accuracy have been proposed. Emerging solutions employ transfer learning frameworks (Bian et al., 2023; Gao et al., 2023c) to minimize calibration in noninvasive systems, while invasive BCIs benefit from inherent signal fidelity to achieve rapid calibration (Huang et al., 2021). A recent study has demonstrated the potential for real-time control of prosthetic devices using invasive EEG-BCIs, highlighting their potential for clinical applications (AlQahtani et al., 2024). Calibration is generally more efficient for minimally invasive EEG-BCIs than noninvasive systems; nonetheless, it is less risky than for invasive systems (Huang et al., 2021). Recent research has focused on developing hybrid approaches that combine the benefits of both noninvasive and invasive techniques.

## Paradigms

Experimental paradigm guides the generation of specific neural signals through task design, thereby shaping the EEG acquisition process in reverse. In addition, its design critically determines the operational logic and classification basis of EEG-based BCIs. Given the inherently concurrent and complex nature of neural oscillations, isolating specific brain activity patterns necessitates strategic paradigm implementation during signal acquisition. The paradigm acts as a neural encoder, encoding cortical activity to elicit distinguishable electrophysiological signatures and provide a physical basis for decoding algorithms.

Over the decades, diverse BCI paradigms have emerged to target distinct neural correlates. Some paradigms are specifically engineered to provoke motor-related brain activities, exemplified by the MI paradigm (Al-Saegh et al., 2021). External stimulation paradigms employ visual, auditory, or tactile stimuli to evoke event-related potentials for device control (Abiri et al., 2019).

The systematic evolution of these paradigms has propelled BCI technology toward finer-grained brain-state discrimination and more reliable system control.

### Motor imagery paradigms

MI has emerged as a predominant paradigm in BCI research due to its adaptability across applications and demonstrated performance enhancements (Al-Saegh et al., 2021). MI involves the cognitive simulation of physical movements without actual motor execution or muscular activation. Current literature primarily documents two principal MI paradigms, namely SMR and IBK (Abiri et al., 2019; Saibene et al., 2023).

#### Sensorimotor rhythm paradigms

The SMR paradigm represents one of the most established approaches in MI research. This methodology focuses on the mental rehearsal of kinesthetically intensive movements involving major body parts (e.g., hands, feet, or tongue), which induces measurable modulations in cortical activation patterns (Morash et al., 2008).

**Figure 7:**
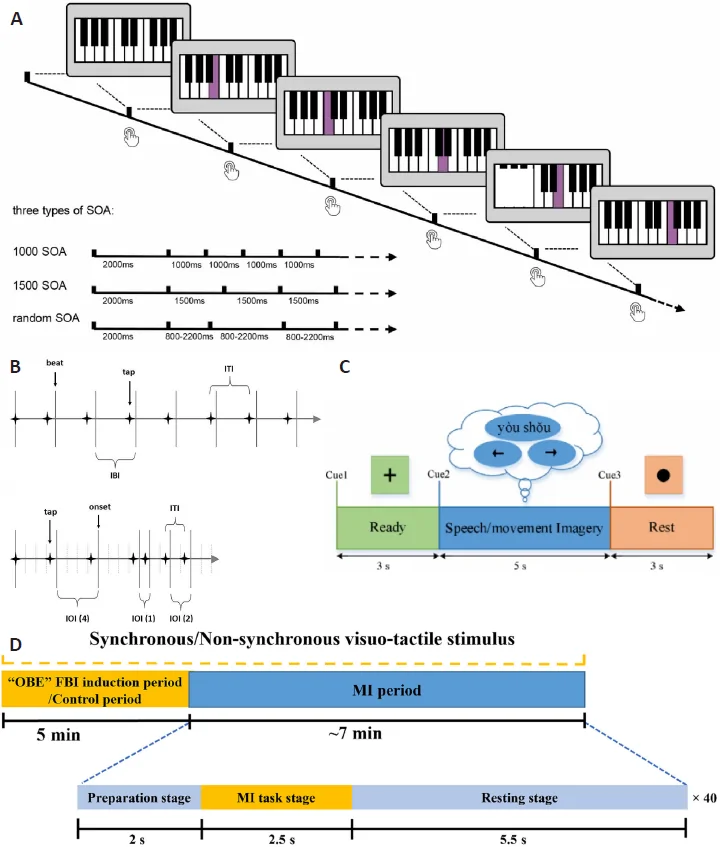
Examples of SMR paradigms for EEG-BCI. (A) Design of a new BCI encoding paradigm that uses rhythmic temporal prediction to enhance the detection of movement intention. Reprinted from Meng et al. (2023b). Copyright 2023, IOP Publishing. (B) Design of a test with mixed stimuli from the tempo and rhythm subtests of the profile of music perception skills. Reprinted from Georgi et al. (2022). (C) Design of a novel MI paradigm that integrates speech imagery with silent reading and writing imagery. Reprinted from Tong et al. (2023). (D) Design of a novel paradigm for enhancing MI from the third-person perspective. Reprinted from Xu et al. (2024). Copyright 2024, Federation of European Neuroscience Societies and John Wiley & Sons Ltd. BCI: Brain–computer interface; EEG: electroencephalography; FBI: full-body illusion; MI: motor imagery; IBI: interbeat interval; IOI: intereset interval; ITI: intertap interval; OBE: out-of-body experience; SMR: sensorimotor rhythms; SOA: stimulus onset asynchrony.

Recently, a rhythmic temporal prediction paradigm (**Figure 7A**) was proposed to enhance the detection of movement intention (Meng et al., 2023b). The 1000 ms prediction condition demonstrated greater beta event-related desynchronization (ERD) lateralization in the motor area (*P* < 0.001), larger beta ERD in the frontal area (*P* < 0.001), and higher decoding accuracy (averaged left–right decoding accuracy of 89.71% using the common spatial pattern and 97.30% using Riemann tangent space), suggesting promising applications for motor-based BCIs. A mixed stimulus test from the tempo and rhythm subtests of the profile of music perception skills (**Figure 7B**) was designed to assess the ability to tap into complex rhythmic patterns (Georgi et al., 2022). Administered as tapping items to 40 participants, the results demonstrated satisfactory internal consistency (McDonald’s omega of 0.44 and 0.58 for absolute and relative rhythm synchrony subtests) and test–retest reliability (intraclass correlation coefficients of 0.90 and 0.85, Spearman’s rank correlation coefficients of 0.78 and 0.74 for tempo synchrony and the number of correctly reproduced rhythm items). This tool assesses complex rhythmic tapping skills in 15 minutes, complementing existing music aptitude batteries.

#### Imagined body kinematics paradigms

While SMR-based approaches have dominated MI research, investigators have increasingly focused on extracting detailed kinematic information from EEG signals (McFarland et al., 2010). Unlike SMR paradigms that detect gross motor intentions, IBK paradigms aim to decode specific movement parameters (e.g., velocity, acceleration, spatial trajectory) through low-frequency SMR signals (< 2 Hz) adapted from invasive BCI technology (Hochberg et al., 2006; Kim et al., 2008; Yuan and He, 2014; Pereira et al., 2017). This paradigm employs distinct training protocols emphasizing 3D mental simulation of isolated body part movements, with time-domain signal decoding aligned with naturalistic motion imagery.

A novel MI paradigm was proposed to enhance BCI performance by integrating speech imagery with silent reading of Chinese Pinyin phonetic transcriptions and writing imagery of Chinese characters (**Figure 7C**; Tong et al., 2023). In comparative experiments, the proposed paradigm achieved 77.03% classification accuracy, 9.07% higher than that observed with the traditional MI paradigm (68.96%). These results show that the proposed paradigm elicited more discriminative EEG features, improving classification performance and highlighting multimodal paradigms as a novel approach for robust BCI feature extraction. An innovative virtual reality-enhanced (VR-enhanced) MI paradigm (**Figure 7D**) leveraging third-person embodiment through visuo-tactile stimulation was presented (Xu et al., 2024). By inducing sense of body ownership via synchronized multisensory input, the protocol significantly strengthened SMR ERD in 31 healthy participants compared to conventional first-person MI approaches. These findings validate VR-mediated sense of body ownership manipulation as an effective strategy for MI enhancement, particularly for rehabilitation applications requiring perspective-shifting training modalities.

### External stimulation paradigms

External stimuli such as flashing lights and auditory cues can affect brain activity, which can be recorded and decoded to enable device or prosthetic control. This is the core of external stimulation paradigms, including visual (Kilmarx et al., 2024; Zhang et al., 2024a), auditory (Borirakarawin and Punsawad, 2023; Zhang et al., 2024a), and somatosensory (Savić et al., 2023; Isenstein et al., 2024) paradigms. Notable examples include the SSVEP paradigm (Chailloux Peguero et al., 2023; Niu et al., 2023) and the visual P300 paradigm (Demirayak et al., 2023; Ranjan et al., 2024).

#### Steady-state visually evoked potential paradigms

The SSVEP paradigm, a widely adopted EEG-based BCI method, leverages rhythmic visual stimuli to elicit frequency-locked neural oscillations in the primary visual cortex. Its popularity stems from its capacity to achieve high performance through calibration-dependent recognition algorithms (Xu et al., 2021).

**Figure 8:**
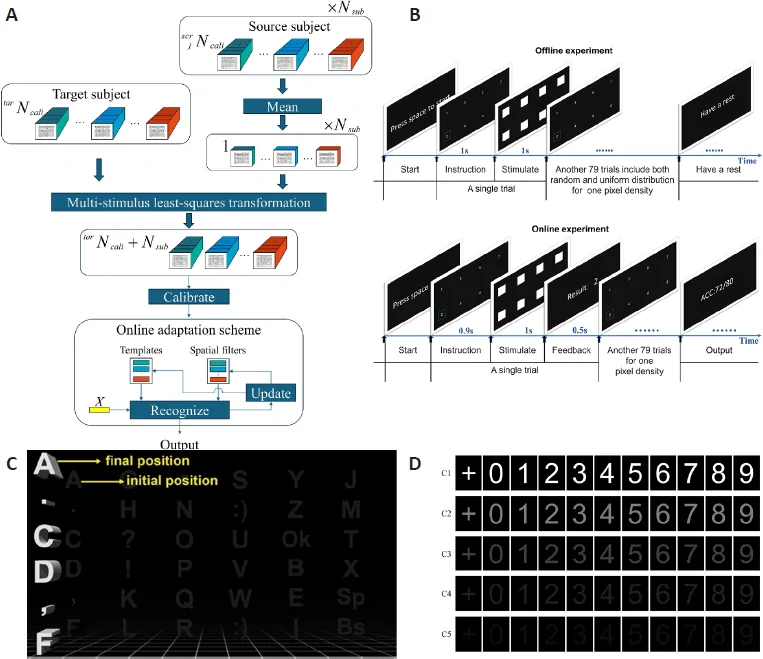
Examples of external stimulation paradigms for EEG-BCIs. (A) Design of the ms-LST-OA. Reprinted from Li et al. (2024b). (B) Design of a novel low-pixel-density encoding paradigm for SSVEP-based BCIs. Reprinted from Meng et al. (2023a). (C) Design of a 3D column P300 stimulus presentation paradigm. Reprinted from Korkmaz et al. (2023). (D) Design of an RSVP paradigm under five levels of weak hidden conditions. Reprinted from Lian et al. (2023). 3D: Three-dimensional; BCI: brain‒computer interface; EEG: electroencephalography; ms-LST-OA: multistimulus least-squares transformation with an online adaptation scheme; RSVP: rapid serial visual presentation; SSVEP: steady-state visually evoked potential.

A multi-stimulus least-squares transformation with an online adaptation scheme (**Figure 8A**) was presented to reduce calibration effort for SSVEP-based BCIs (Li et al., 2024b). By integrating cross-stimulus learning to refine transformation matrices and optimizing spatial filters and reference templates via online adaptation, the multi-stimulus least-squares transformation with an online adaptation scheme achieved impressive information transfer rates (ITRs) of 210.01 ± 10.10, 172.31 ± 7.26, and 139.04 ± 14.90 bits/min across three datasets, using minimal calibration data. A novel low-pixel-density encoding paradigm (**Figure 8B**) was proposed for SSVEP-based BCIs, aiming to balance user comfort and system effectiveness (Meng et al., 2023a). The results of three experiments comparing presentation forms (flickering squares *vs.* checkerboards), pixel distribution patterns (random *vs.* uniform), and pixel densities (20%–100%) confirmed the feasibility of 60%-density stimuli in real-time BCI applications, demonstrating both user comfort and robust system effectiveness. This innovation advances the development of naturalistic and ergonomic BCI applications.

#### Visual P300 paradigm

The visual P300, an event-related potential first documented in 1967 (Sutton et al., 1967), was adapted for BCI applications by Farwell and Donchin (1988). Their foundational row‒column paradigm employed a 6 × 6 matrix to evoke P300 responses via randomized row/column highlighting. While slower than conventional methods do, this approach has proven vital for communication-impaired individuals. Cuntai et al. (2004) introduced the single display speller, which isolated character highlighting to reduce flash counts but amplify P300 amplitudes. Later innovations included the region-based paradigm, which uses dual-stage target identification (Fazel-Rezai and Abhari, 2009); the submatrix-based paradigm, which divides row‒column paradigm matrices into submatrices (Shi et al., 2012); and the rapid serial visual presentation (RSVP) paradigm, which presents sequential characteristics at a fixed gaze-independent location (Acqualagna et al., 2010; Acqualagna and Blankertz, 2013).

Recently, a novel probabilistic and 3D column P300 stimulus presentation paradigm (**Figure 8C**) was presented for EEG-based spelling systems (Korkmaz et al., 2023). Diverging from traditional 2D row‒column designs, this approach adaptively adjusts column flash counts on the basis of dictionary-derived letter probabilities. The experimental results demonstrated a 92.69% average classification accuracy—9.94% higher than nonprobabilistic 3D paradigms—underscoring its potential for high-performance BCI spelling. An RSVP paradigm (**Figure 8D**) was presented for EEG-based target detection under five weak hidden conditions defined via RGB color space metrics (Lian et al., 2023). In 18 participants, the experimental results revealed that the P300 amplitude decreased (8.92 ± 1.24 µV *vs*. 7.84 ± 1.40 µV, *P* = 0.021) and the latency increased (582.39 ± 25.02 ms *vs.* 643.83 ± 26.16 ms, *P* = 0.028) under the weakest hidden condition. Notably, when the proposed channel selection method was used, the detection accuracy decreased minimally (75.04% ± 3.24% *vs*. 73.35% ± 3.15%, *P* = 0.029) despite a > 90% reduction in channels (62 *vs*. 6) under the same conditions, confirming the feasibility of EEG-based target detection in resource-constrained BCI environments.

### Hybrid paradigms

Hybrid paradigms integrate two or more physiological signals, with EEG as a mandatory component (Pfurtscheller et al., 2010; Amiri et al., 2013). In BCI research, such paradigms may involve the fusion of multiple BCI approaches or the combination of EEG with supplementary physiological signals. These auxiliary signals commonly include EMG, ECG, electrooculography (EOG), and hemodynamic responses measured via fNIRS.

#### Combination of brain–computer interface paradigms

Recent advancements in hybrid BCI systems demonstrate that combining distinct paradigms significantly improves performance. Visual P300, SSVEP, and SMR paradigms are the most commonly used paradigms in the development of hybrid BCI systems (Kapgate and Kalbande, 2015).

**Figure 9:**
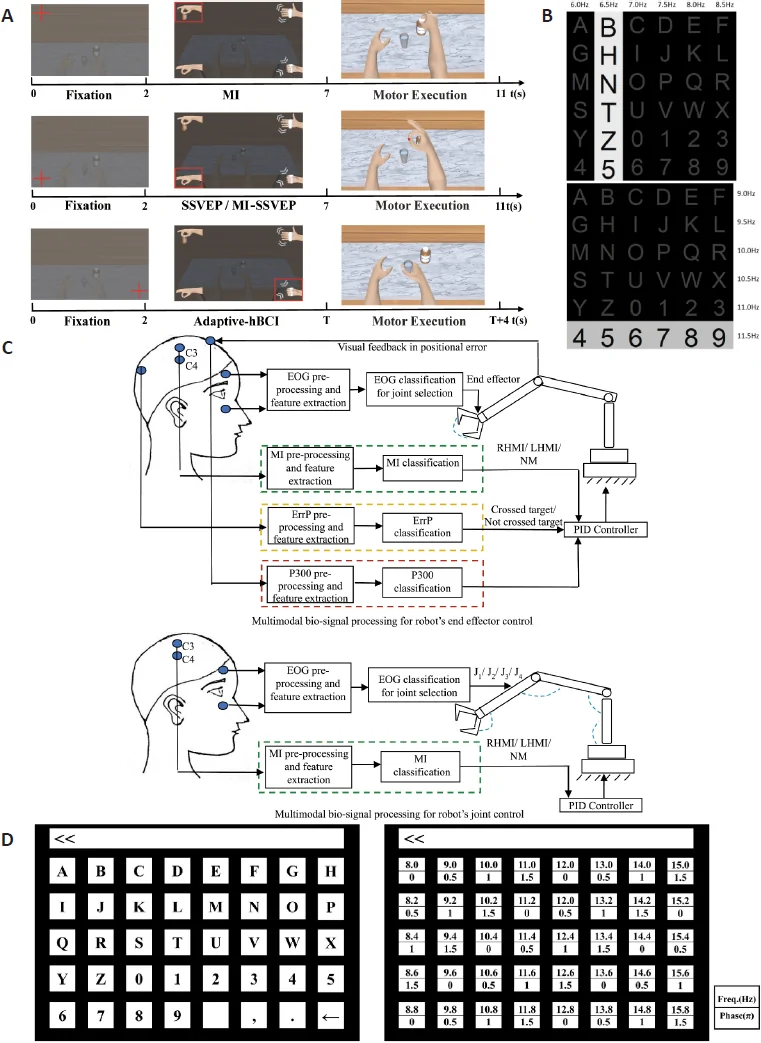
Examples of hybrid paradigms for EEG-BCI. (A) The paradigm design of an adaptive hybrid BCI system combining the MI and SSVEP paradigms. Reprinted from Su et al. (2024). (B) The paradigm design of a novel hybrid BCI speller system that integrates P300 and SSVEP signals. Reprinted from Bai et al. (2023). (C) The paradigm design of a novel hybrid BCI system that integrates EEG and EOG signals to control a robotic arm. Reprinted from Ghosh et al. (2025). Copyright 2024, Elsevier B.V. (D) The paradigm design of a hybrid BCI system that combines MEG and EEG. Reprinted from Li et al. (2023c). BCI: Brain–computer interface; EEG: electroencephalography; EOG: electrooculography; ErrP: error-related potential; LHMI: left-hand motor imagery; MEG: magnetoencephalography; MI: motor imagery; NM: no movement; PID: proportional integral derivative; RHMI: right-hand motor imagery; SSVEP: steady-state visually evoked potential.

Recently, an adaptive hybrid BCI system (**Figure 9A**) combining MI and SSVEP was proposed for hand function rehabilitation in patients who had a stroke (Su et al., 2024). This calibration-free system employs a VR-based flicker coding method, integrating real-time ERD monitoring with dual-task interference, and using a soft robotic glove for physical therapy. The system demonstrated high four-gesture recognition accuracies (94.37% ± 4.77% for healthy individuals and 79.38% ± 6.26% for patients who had a stroke) and neural activation in both healthy individuals and patients who had a stroke, showing potential to promote neuroplasticity and functional recovery. A hybrid BCI speller combining P300 and SSVEP signals (**Figure 9B**) was presented (Bai et al., 2023). Its frequency-enhanced row and column paradigm assigns specific flicker frequencies to a 6 × 6 character matrix, enabling simultaneous elicitation of P300 and SSVEP responses. Online testing achieved an average accuracy of 94.29% and an ITR of 28.64 bits/min, outperforming single-paradigm P300 or SSVEP spellers. This work underscores the practical value of hybrid paradigms for enhancing BCI communication systems.

#### Brain–computer interface paradigms based on electroencephalography and other physiological signals

To address inherent limitations of EEG signals—including low amplitude, nonstationarity, and susceptibility to muscle artifacts—researchers increasingly adopt multimodal approaches incorporating EMG, EOG, or ECG. These hybrid systems enhance data robustness and reliability for neuroscience applications (Choi et al., 2017).

A novel hybrid BCI system (**Figure 9C**) that integrates EEG and EOG was proposed to control a robotic arm (Ghosh et al., 2025). In this system, an EOG and an EEG sensor have been used to select robot joints and control their directional movement, respectively. The classifiers achieve 98.45% (EOG) and 96.61% (EEG) accuracy, enabling real-time online robot navigation. The framework features a 0.036 average steady-state error, 2.5% peak overshoot, 30 second settling time, and 95% average target reaching rate, making it suitable for real-time rehabilitative prosthetics platforms. A hybrid magnetoencephalography-EEG (MEG-EEG) system (**Figure 9D**) was proposed to improve steady-state visual evoked response-based BCIs (Li et al., 2023c). Through comparative experiments with 22 subjects, the investigators demonstrated that MEG-derived steady-state visual evoked responses exhibit a broader effective bandwidth and higher SNR. Hybrid MEG-EEG fusion achieved a 28% higher ITR (312 ± 17 bits/min) compared to standalone EEG (240 ± 27 bits/min), and 15% improvement over MEG alone (272 ± 17 bits/min). Crucially, the hybrid system elevated 40-target classification accuracy for “BCI illiterate” users from 50% to 95%, effectively mitigating performance variability. These findings establish multimodal neuroimaging as a viable paradigm for developing robust, high-speed BCIs while providing mechanistic insights into cross-modality neural feature complementarity.

### Conclusion and prospects

**Additional Table 2** summarizes the advantages and disadvantages of MI, SSVEP, P300, and hybrid paradigms.

**Additional Table 2:** The advantages and disadvantages of the MI, SSVEP, P300, and hybrid paradigms

Paradigm	Advantage	Disadvantage
MI	• Inherent discriminative properties for task classification • Synergizes with physical therapy for stroke rehabilitation • Mature signal processing techniques with extensive research foundation	• Requires weeks to months of training for 2D/3D cursor control • Relies on voluntary modulation of neural frequency bands • Limited practical application of IBK
SSVEP	• No user training needed, broad applicability • Multifrequency flickering enables diverse commands • Superior classification reliability compared to ERP	• Flickering stimuli cause visual fatigue (especially at low frequencies) • Incompatible with visually impaired users • Lack of standardized stimulus parameters; signal quality depends on stimulation duration
P300	• Rapid calibration • High accuracy and typing speed improvements • Suitable for immediate device control	• High attentional demands induce user fatigue • Visual dependency excludes visually impaired populations • Declining satisfaction during prolonged use
Hybrid	• Enhanced performance via multimodal integration	• Increased complexity raises hardware/algorithmic demands, risking real-time performance
paradigm	• Reduced false positives • Supports complex control and communication • Balances exogenous and endogenous signals to optimize training effort and control efficiency	• Concurrent attention to multiple feedback modes elevates cognitive load • Extended calibration • Technical incompatibility

2D: Two-dimensional; 3D: three-dimensional; EEG: electroencephalography; ERP: evoked event-related potential; IBK: imagined body kinematics; MI: motor imagery; SSVEP: steady- state visually evoked potential.

The MI paradigm, as previously discussed, is distinguished by several strengths that position it as a compelling methodology in BCI research (Baig et al., 2017; Padfield et al., 2019). Its natural connection to movement planning creates distinct brainwave patterns that simplify system training while proving particularly effective in stroke recovery programs when combined with traditional physical therapy (Cheng et al., 2018). The substantial research infrastructure surrounding MI-BCIs has driven significant progress in neural signal decoding methodologies, further amplifying its clinical potential. However, critical limitations persist. The primary constraint of the SMR paradigm lies in its extended training requirements—often spanning weeks to months—to achieve proficient 2D/3D cursor control. This process necessitates subjects to volitionally modulate specific spectral components of neural oscillations for directional cursor navigation during target acquisition tasks (Abiri et al., 2019). While contemporary MI developments predominantly employ IBK, their operational implementation in practical BCI systems remains constrained, warranting further investigation.

SSVEP paradigms offer distinct operational advantages in both neuroprosthetic applications and cognitive neuroscience research. As an exogenous-driven approach, it requires no subject training while maintaining broad user compatibility. The ability to encode multiple commands through frequency-specific visual flickering enables enhanced prosthetic controllability, complemented by superior classification reliability compared to event-related potential paradigms. However, technical and physiological challenges include user fatigue from prolonged flicker exposure (particularly at lower frequencies) and reduced accessibility for visually impaired populations due to gaze-dependent operation (Chang et al., 2014). Additional limitations encompass variability in stimulus parameter standardization across studies and temporal modulation effects on SSVEP signal integrity (Vialatte et al., 2010). Current research initiatives aim to optimize stimulation protocols and expand neurological applications while mitigating these constraints.

The P300 speller paradigm has achieved notable progress in enhancing classification accuracy and character throughput. Its principal advantage resides in rapid system calibration—typically requiring minutes—enabling near-immediate user operability. Despite iterative improvements in interface ergonomics, persistent issues include cognitive fatigue stemming from sustained visuospatial attention demands during extended operation (Fazel-Rezai et al., 2012), along with inherent accessibility barriers for visually impaired users (McCane et al., 2014). Future developmental research efforts should focus on adaptive optimization of attentional load parameters and multimodal feedback integration to improve user comfort and inclusivity.

Hybrid BCI paradigms, which integrate multiple physiological signals, offer significant advantages and challenges. Key strengths include enhanced performance (e.g., classification accuracy, robustness, and ITR) through complementary signal utilization and leveraging synergies between exogenous and endogenous signals. Hybrid systems also reduce false-positive rates via cross-modal validation and support complex applications, such as neuroprosthetics or robotic rehabilitation. However, increased system complexity arises from synchronizing heterogeneous signal sources, leading to hardware integration challenges, latency issues, and higher computational demands. User cognitive load escalates due to simultaneous engagement with multiple feedback modalities (e.g., visual attention and motor intent), potentially causing fatigue (Ahn et al., 2024). Additionally, extended calibration times and technical compatibility hurdles (e.g., signal synchronization between EEG and eye-tracking devices) limit practical deployment. Despite these barriers, hybrid paradigms hold promise for advancing multi-degree-of-freedom control and adaptive neurorehabilitation, contingent on optimizing algorithmic efficiency and user-centered design.

## Decoding Algorithms

Decoding algorithms serve to interpret underlying neural processes from recorded electrophysiological signals, convert them into control commands and, thereby, facilitate practical applications. Moreover, advancements in decoding algorithms can also drive forward the development of EEG electrode technology and the refinement of encoding paradigms. While experimental paradigms are intentionally designed to encode targeted neural activity within complex brain signal mixtures, the inherently low SNR of EEG measurements presents challenges for effective decoding using conventional machine learning approaches. Furthermore, the substantial variability in EEG patterns induced by different experimental paradigms necessitates the development of paradigm-specific decoding architectures. To address these challenges, researchers have developed sophisticated signal decomposition techniques for feature extraction, exemplified by the successful application of common spatial pattern analysis in MI paradigms. Contemporary EEG-based BCI decoding frameworks primarily fall into three categories, namely Riemannian geometry, deep learning architectures employing neural networks for automated feature learning, and transfer learning (Xu et al., 2021).

### Riemannian geometry

Over the past decade, Riemannian geometry-based methodologies—pioneered through the application of covariance matrices—have evolved into a prominent analytical framework for BCI research, garnering increasing adoption within the scientific community. These approaches have gained prominence in BCI research due to their algorithmic simplicity, computational efficiency, and robust classification accuracy. A typical Riemannian BCI pipeline comprises three stages, namely signal preprocessing, feature extraction, and classification (Yger et al., 2017).

#### Preprocessing

The preprocessing of EEG data serves to mitigate the stochastic elements within the signals, thereby preserving the salient information (Rashid et al., 2020). This stage addresses three critical challenges: mitigating noise (e.g., environmental interference); suppressing physiological artifacts (e.g., ocular/muscular activity); and enhancing discriminative features to optimize classification performance.

**Figure 10:**
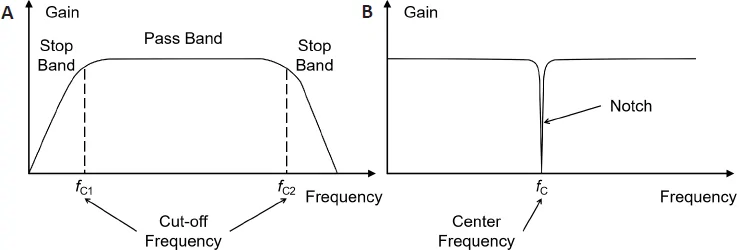
Schematic diagram of the filter. (A) Bandpass filter. (B) Notch filter.

Spectral filtering constitutes a cornerstone of preprocessing, given the neurophysiological relevance of alpha (8–13 Hz) and beta (14–30 Hz) rhythms in MI, cognitive processing, and sensorimotor integration tasks where ERD and event-related synchronization phenomena occur (Yger et al., 2017; Kumar et al., 2019; Zhidong et al., 2020). While conventional asynchronous BCI pipelines typically employ both spatial and frequency filtering (Barachant et al., 2010), Riemannian approaches uniquely circumvent the need for spatial filtering (Barachant et al., 2012; Kumar et al., 2019). Key preprocessing steps include band-pass filtering (**Figure 10A**) that isolates frequency bands of interest, notch filtering (**Figure 10B**) that eliminates line noise (e.g., 50/60-Hz interference), and epoching that segments continuous EEG into task-specific temporal windows, typically aligned to visual/auditory cues triggering MI or motor execution tasks (Shuqfa et al., 2023). To identify the period during which event-related synchronization and ERD occur, numerous researchers epoch EEG trials during preprocessing.

#### Feature extraction

Feature extraction and classification form the computational core of BCI systems (Tibermacine et al., 2024), requiring precise identification of neurophysiological patterns that reflect user intent. Traditional methodologies include common spatial pattern, wavelet transform, adaptive regression model, and connectivity features such as directed transfer function, spectral coherence, or phase locking values (Lotte et al., 2018; Rodrigues et al., 2019; Singh et al., 2019; Zhidong et al., 2020; Pandey et al., 2021; Shuqfa et al., 2023). These methods are increasingly being supplemented or outperformed by Riemannian geometry-based approaches operating on covariance matrices in symmetric positive definite space.

**Figure 11:**
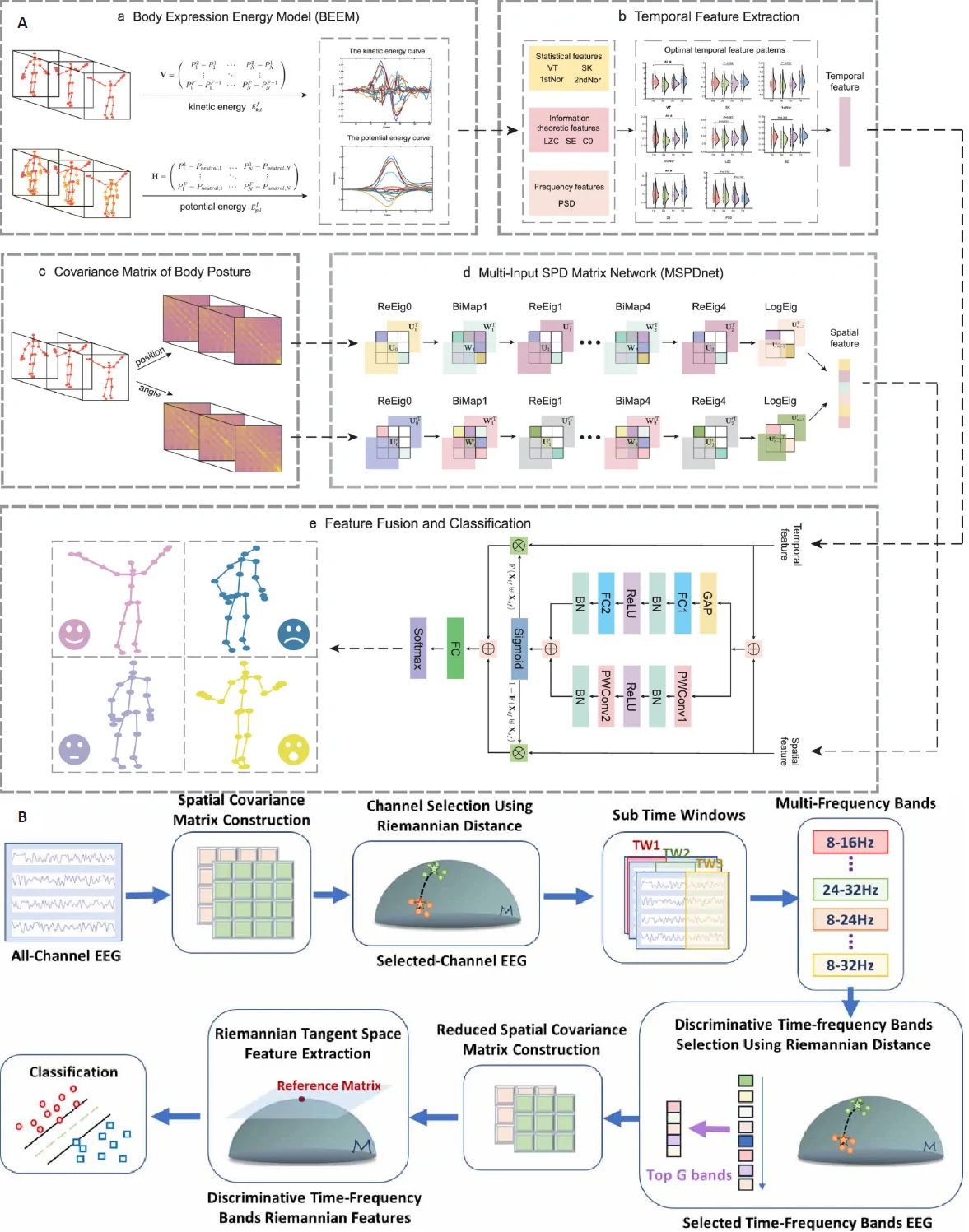
Examples of Riemannian geometry-based decoding algorithms used for feature extraction in EEG-BCI. (A) Overview of the Riemannian manifold-based framework presented for emotion recognition through body expression analysis. Reprinted from Wang et al. (2025). Copyright 2024, Elsevier B.V. (B) Overview of a method presented to enhance MI-BCIs through Riemannian manifold-driven channel selection and tangent space feature extraction. Reprinted from Qu et al. (2022). Copyright 2022, IOP Publishing. 1stNor: First differences; 2ndNor: second differences; BN: batch normalization; C0: C0 complexity; FC: fully connected; GAP: global average pooling; LZC: Lempel–Ziv complexity; PSD: power spectral density; SE: Shannon entropy; SK: skewness; SPD: symmetric positive definite; VT: variance trend.

Recently, a Riemannian manifold-based framework (**Figure 11A**) was presented for emotion recognition through body expression analysis (Wang et al., 2025). It employed a multi-input symmetric positive definite matrix network, capturing spatial Riemannian relationships between body joints. The attentional temporal-spatial feature fusion algorithm was introduced to fuse temporal and spatial features, thus enhancing discriminability across emotional states. The proposed method achieves recognition accuracies of over 90% across four public datasets, outperforming most state-of-the-art approaches. Riemannian geometry was used for classifying error-related potentials in BCIs (Tang et al., 2021). The researchers conducted an experiment with seven participants performing a visual discrimination task, during which they recorded multi-channel EEG to classify error-related potentials. Results demonstrated the superiority of Riemannian methods (78.2% accuracy) over traditional time-domain features (75.9%), highlighting their utility for BCI error correction without laborious feature engineering. Riemannian procrustes analysis was employed to achieve EEG-based cognitive load prediction, identifying the covariance matrices of EEG signal derivatives as optimal features (Kremer et al., 2024). This analysis demonstrated that models using these features outperform existing ones, with the performance evaluated through Riemannian procrustes analysis on subjects not included in the training dataset. The research proves that Riemannian procrustes analysis is a promising method for generalizing across subjects, particularly in applications with limited calibration time. A method (**Figure 11B**) was presented to enhance MI-BCIs through Riemannian manifold-driven channel selection and tangent space feature extraction from discriminant spectral bands (Qu et al., 2022). This approach achieved high accuracy on two public BCI datasets (90.0% and 89.4% for 16-channel EEG signals), showing promise for improving MI-BCI performance.

#### Classification

Riemannian classifiers in BCI applications primarily operate through two methodological approaches, namely direct manifold implementation (as observed in Riemannian minimum distance to mean) or via tangent space projection (Lotte et al., 2018).

**Figure 12:**
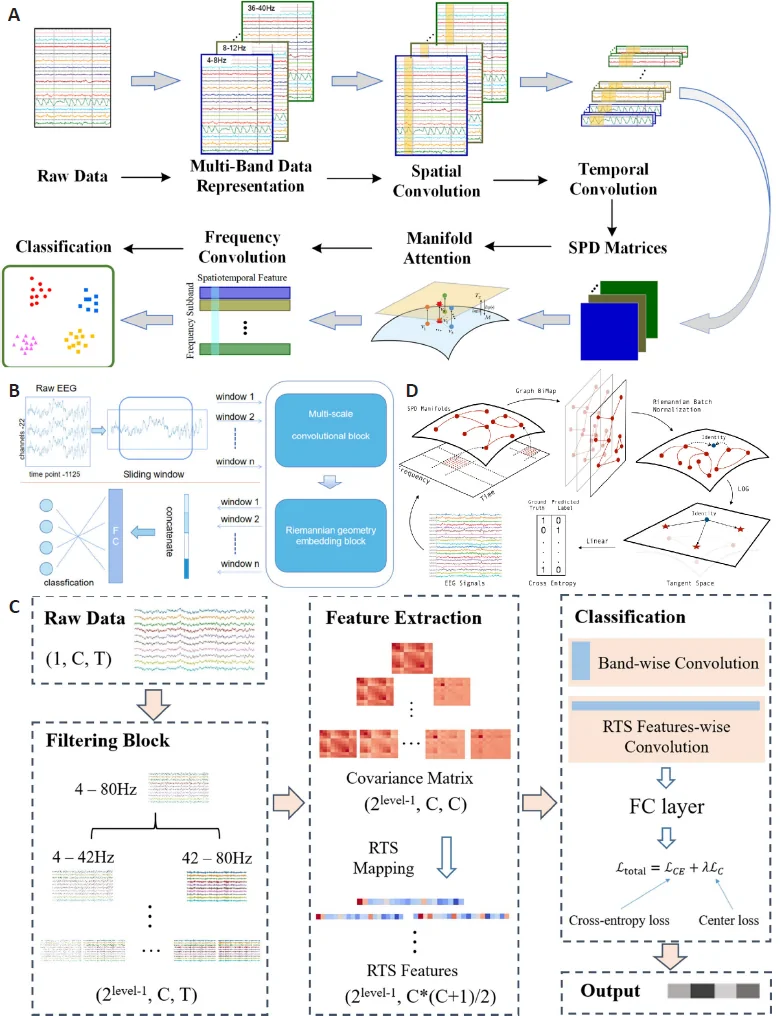
Examples of Riemannian geometry-based decoding algorithms used for classification in EEG-BCI. (A) The overall framework of the MAMCNet. Reprinted from Lu et al. (2024). Copyright 2024, Elsevier B.V. (B) The overall framework of MSCARNet. Reprinted from Zhou et al. (2024). (C) The overall framework of the DFBRTS. Reprinted from Xiong et al. (2025). Copyright 2024, Elsevier Ltd. (D) The overall framework of the Graph-CSPNet. Reprinted from Ju and Guan (2024). BCI: Brain–computer interface; DFBRTS: Riemann tangent space mapping using a dichotomous filter bank with a convolutional neural network; EEG: electroencephalography; FC: fully connected; MAMCNet: manifold attention-based multidomain convolutional neural network; MSCARNet: multiscale convolutional attention and Riemannian geometry network; RTS: Riemann tangent space; SPD: symmetric positive definite.

Research demonstrates that basic Riemannian geometry-based classifiers operating on the manifold exhibit competitive performance compared to conventional state-of-the-art BCI classifiers, particularly in systems with moderate electrode counts. These classifiers demonstrate notable noise robustness and enhanced generalization capabilities, validated across both healthy cohorts and clinical populations (Barachant et al., 2012; Kalunga et al., 2016; Mayaud et al., 2016). In recent years, deep learning approaches have been employed for Riemannian geometry-based classifiers on the manifold. A novel manifold attention-based multi-domain CNN (**Figure 12A**) was presented for decoding MI intentions from EEG signals through spatiotemporal refinement on Riemannian manifolds using attention mechanisms (Lu et al., 2024). By leveraging intrinsic information from temporal, spectral, and spatial EEG modalities, manifold attention-based multi-domain CNN outperforms conventional deep learning models across two benchmark datasets (accuracy: 76.05% ± 6.76% and 91.00% ± 6.65%, kappa: 0.68 ± 0.09 and 0.82 ± 0.14), establishing itself as a promising alternative for BCI implementations. A multi-scale convolutional attention and Riemannian geometry network (**Figure 12B**) was presented for MI-EEG classification (Zhou et al., 2024). The network integrates multi-scale convolution with Riemannian geometry and is enhanced by an attention mechanism and sliding window technique. The framework achieves an accuracy improvement of 4% and 2.5% over the existing state-of-the-art method in subject-dependent and subject-independent classification (82.66% *vs*. 78.62% and 61.24% *vs*. 58.74%), respectively. These advancements collectively demonstrate the efficacy of Riemannian space hybridization in boosting EEG classification accuracy.

Tangent space projection methods for Riemannian geometry-based classifiers have shown particular promise, surpassing other state-of-the-art approaches in classification accuracy (Barachant et al., 2012, 2013). This technique typically employs standard classifiers (e.g., linear discriminant analysis, support vector machine, logistic regression) following manifold-to-tangent space mapping. A novel Riemann tangent space mapping using a dichotomous filter bank with CNN (**Figure 12C**) was presented, integrating Riemannian geometry with cross-frequency coupling for MI-EEG decoding (Xiong et al., 2025). Validated on two public datasets, the dichotomous filter bank with CNN achieved superior four-class (78.16%) and two-class (71.58%) classification accuracy, surpassing existing benchmarks and demonstrating significant improvements in robust MI-BCI implementation. A novel approach termed Graph-Cross Stage Partial Network (Graph-CSPNet) (**Figure 12D**), an architecture employing manifold-valued graph convolutional methods, was proposed for time-frequency EEG analysis (Ju and Guan, 2024). Rooted in differential geometry principles from EEG covariance matrices, this framework enhances signal segmentation flexibility and localized variation detection. Evaluation across five public MI-EEG datasets yielded near-optimal classification accuracy in nine of 11 experimental scenarios, establishing new benchmarks for geometric deep learning applications in BCI systems.

### Deep learning

In recent years, deep learning has witnessed a remarkable surge in applications across diverse disciplines. As an advanced subset of machine learning, it has demonstrated exceptional capabilities in fields such as computer vision, revolutionizing the approach through which machines analyze and interpret images and videos. Beyond these domains, deep learning has also made significant strides in BCI research, marking a pivotal advancement toward more intuitive and efficient brain-computer interactions. Currently, the deep learning models predominantly utilized in EEG-based BCI decoding algorithms include CNNs, RNNs, AEs, DBNs, transformers, and hybrid architectures.

#### Convolutional neutral network

Among all the deep learning architectures for EEG-based BCI decoding, the CNN is the most prevalent architecture design framework (Craik et al., 2019). Many CNN varieties have been used in decoding algorithms, including attention-based CNNs (Fan et al., 2021; Tao et al., 2024), inception-based CNNs (Zhang et al., 2021b; Ke et al., 2024), residual-based CNNs (Fan et al., 2021; Liu and Yang, 2021; Gao et al., 2023a), DenseNets (Alwasiti et al., 2020; Yang et al., 2023), 3D CNNs (Zhao et al., 2019; Liu and Yang, 2021; Fan et al., 2024), multibranch CNNs (Zhao et al., 2019; Cai et al., 2024b), multilayer CNNs (Amin et al., 2019; Jeong et al., 2020), and multiscale CNNs (Gao et al., 2023a; Wang et al., 2024e).

**Figure 13:**
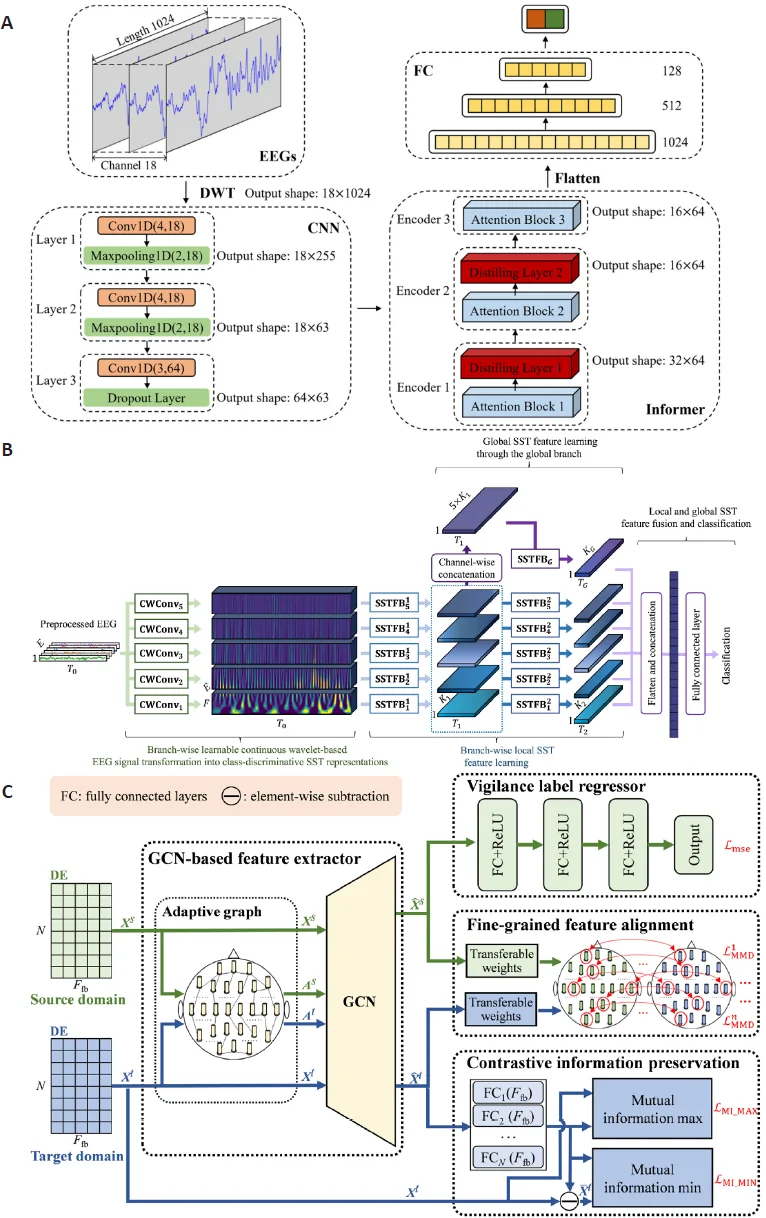
Examples of CNN-based decoding algorithms for EEG-BCI. (A) Overview of CNN-Informer. Reprinted from Li et al. (2025). Copyright 2024, Elsevier Ltd. (B) Overview of the learnable continuous wavelet-based multibranch attentive CNN framework. Reprinted from Kim et al. (2024). Copyright 2024, Elsevier Ltd. (C) Overview of the CFGDAN. Reprinted from Wang et al. (2024c). Copyright 2024, Elsevier Ltd. BCI: Brain–computer interface; CFGDAN: contrastive fine-grained domain adaptation network; CNN: convolutional neural network; CWConv: continuous wavelet-based convolutional layer; DWT: discrete wavelet transform; EEG: electroencephalography; FC: fully connected; GCN: graph convolution network; SSTFB: spatiotemporal–temporal feature extraction block.

Recently, CNN-Informer (**Figure 13A**), a novel end-to-end model, was proposed for automatic seizure detection on long-term EEGs (Li et al., 2025). This architecture integrates CNNs to extract localized features from multichannel EEG signals and Informer, an attention-based architecture renowned for its efficient long-sequence time series forecasting capabilities, to capture long-range temporal dependencies. The experimental results demonstrate the superiority of the CNN-Informer and its strong generalization ability across two EEG datasets. It achieves average sensitivity, specificity, and accuracy of 99.54%, 98.55%, and 98.54%, respectively, on the CHB-MIT dataset. For the SH-SDU dataset, the metrics reach 95.40%, 92.92%, and 92.78%, respectively. In addition, the lightweight architecture of the CNN-Informer makes it suitable for real-time implementation. A learnable continuous wavelet-based multibranch attentive CNN framework was presented for decoding MI-EEG signals (**Figure 13B**; Kim et al., 2024). By automating spatiotemporal feature extraction within subject-specific subbands, the proposed method, with a mean accuracy of 85.47%, outperforms state-of-the-art models on public datasets, offering a powerful and innovative approach to personalized EEG decoding for BCIs. A contrastive fine-grained domain adaptation network (**Figure 13C**) was proposed for EEG-based vigilance estimation in BCI systems (Wang et al., 2024c). By combining adaptive graph convolution networks with fine-grained alignment and contrastive learning, the contrastive fine-grained domain adaptation network outperforms the other methods. This research contributes to minimizing calibration requirements while enhancing the feasibility of implementing vigilance estimation methodologies in real-world applications.

Recently, binary F1-scores, weighted F1-scores, and areas under the curve of 89.86%, 90.52%, 89.15%, 89.75%, 89.83%, and 95.10% accuracy, brain regions and frequencies, respectively, were used, and parallel branches were utilized for multiscale feature extraction. Specifically, the optimal MultiSincNet achieves spectral property prediction via sinc-convolution layer prediction via the EEG signal Mamba model (Gu and Dao, 2023), a new large language model architecture, was proposed. It integrates the structured state space sequence model to handle long data sequences, enabling the handling of irregularly sampled data, unbounded context, and retained computational efficiency in training and testing. By integrating CNNs’ localized feature extraction capabilities with mamba’s efficient modeling of long-range dependencies, the combined framework seamlessly bridges detailed spatial and temporal pattern analysis and global contextual reasoning, enhancing performance on sequence-based tasks while preserving the computational efficiency inherent to both architectures. Therefore, the CNN-mamba structure is increasingly being applied in the decoding algorithm of EEG-BCI (Guo et al., 2025; Yang and Jia, 2025).

#### Recurrent neural network

RNNs are a class of neural networks designed to process sequential data, including temporal sequences and natural language. Their recurrent nature derives from iterative task execution across sequence elements, where each output depends on prior computations through internal memory mechanisms. This architecture makes RNNs particularly effective for context-dependent tasks requiring historical information retention.

**Figure 14:**
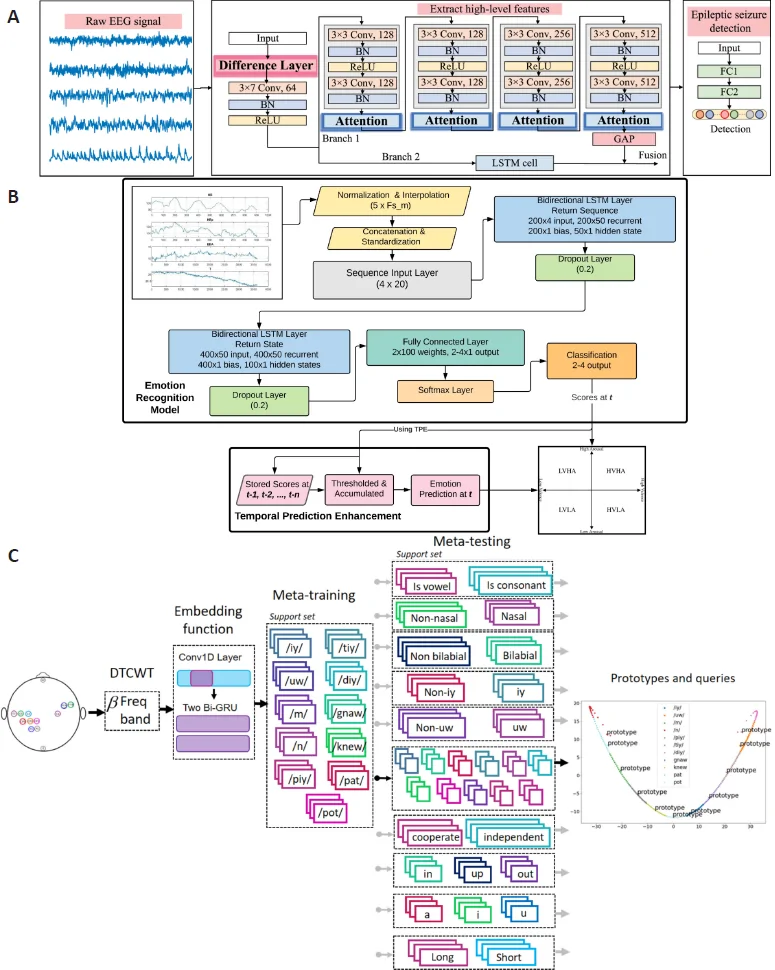
Examples of RNN-based decoding algorithms for EEG-BCI. (A) Overview of the diagram of DARLNet. Reprinted from Qiu et al. (2023). Copyright 2023, Elsevier Ltd. (B) Overview of the emotion recognition model with temporal prediction enhancement. Reprinted from Zitouni et al. (2023). (C) Overview of the Proto-Speech. Reprinted from Hernandez-Galvan et al. (2023). Copyright 2023 Elsevier Ltd. BCI: Brain–computer interface; BN: batch normalization; Conv: convolutional; DTCWT: dual-tree complex wavelet transform; EEG: electroencephalography; FC: fully connected; GAP: lobal average pooling; LSTM: long short-term memory; RNN: recurrent neural network.

Long short-term memory (LSTM), a prominent RNN variant, addresses the vanishing gradient limitation of conventional RNNs through sophisticated gating mechanisms that preserve long-range dependencies. For example, this capability in EEG artifact removal was demonstrated by developing a difference attention network named DARLNet using residual neural network and LSTM to capture spatial correlations and temporal dependencies, respectively (**Figure 14A**; Qiu et al., 2023). Results from comparative experiments demonstrated that DARLNet exhibits superiority on the two-category and five-category epileptic seizure detection tasks, achieving the highest accuracy, precision, recall, and F1-score (98.87%, 98.99%, 99.13%, and 99.06% for two-category task; 90.17%, 90.00%, 90.16%, and 90.05% for five-category task, respectively). A framework for emotion recognition based on the arousal-valence model using multi-modal peripheral physiological signals (**Figure 14B**) was presented (Zitouni et al., 2023). The framework incorporated dynamic thresholding for individual rating variations and introduced a temporal prediction enhancement mechanism to leverage historical predictions, achieving exceptional classification accuracy (> 96% independent and > 93% combined) across diverse experimental scenarios.

Gated recurrent units (GRUs) are a more recent development in the family of RNNs. They simplify the LSTM architecture by combining the forget and input gates into a single “update gate” and removing the cell state, using only a hidden state to store information. This makes GRUs less complex and, in some cases, easier to train. A prototypical network named Proto-Speech (**Figure 14C**) was presented to classify vowels, syllables, and words acquired with the speech imagery paradigm (Hernandez-Galvan et al., 2023). Proto-Speech uses embeddings generated by a one-dimensional convolutional layer and two bidirectional GRU layers to capture spatial and temporal features of EEG signals. The model achieved over 99% accuracy in binary classification tasks and 91.51% in multi-class tasks on the KaraOne dataset, and 93.70% in multiclass evaluation on the ASU dataset, underscoring the efficacy of GRU in processing spatiotemporal EEG features for clinical applications.

#### Auto-encoder

AE is a type of unsupervised feedforward neural network used to learn efficient representations of the input data, typically for dimensionality reduction or feature learning. The fundamental architecture consists of an encoder that maps the input to a latent-space representation and a decoder that reconstructs the input from this representation (Chen and Guo, 2023). At present, there are several different AE models, such as sparse AE (Gao et al., 2024), denoising AE (Vincent et al., 2008), convolutional AE (Masci et al., 2011), and variational AE (Kingma and Welling, 2013).

Key distinctions between these variants emerge from their specialized regularization mechanisms. Sparse AEs introduce sparsity constraints in the loss function, enforcing selective neuron activation to promote efficient data encoding. Denoising AEs enhance feature robustness by training on corrupted inputs, compelling the network to discover noise-invariant hierarchical patterns. Convolutional AEs use CNNs in both encoder and decoder stages, optimizing spatial feature learning through hierarchical downsampling and upsampling operations. Variational AEs adopt a probabilistic framework, employing variational inference to learn latent distributions that enable generative capabilities through optimization of the evidence lower bound.

**Figure 15:**
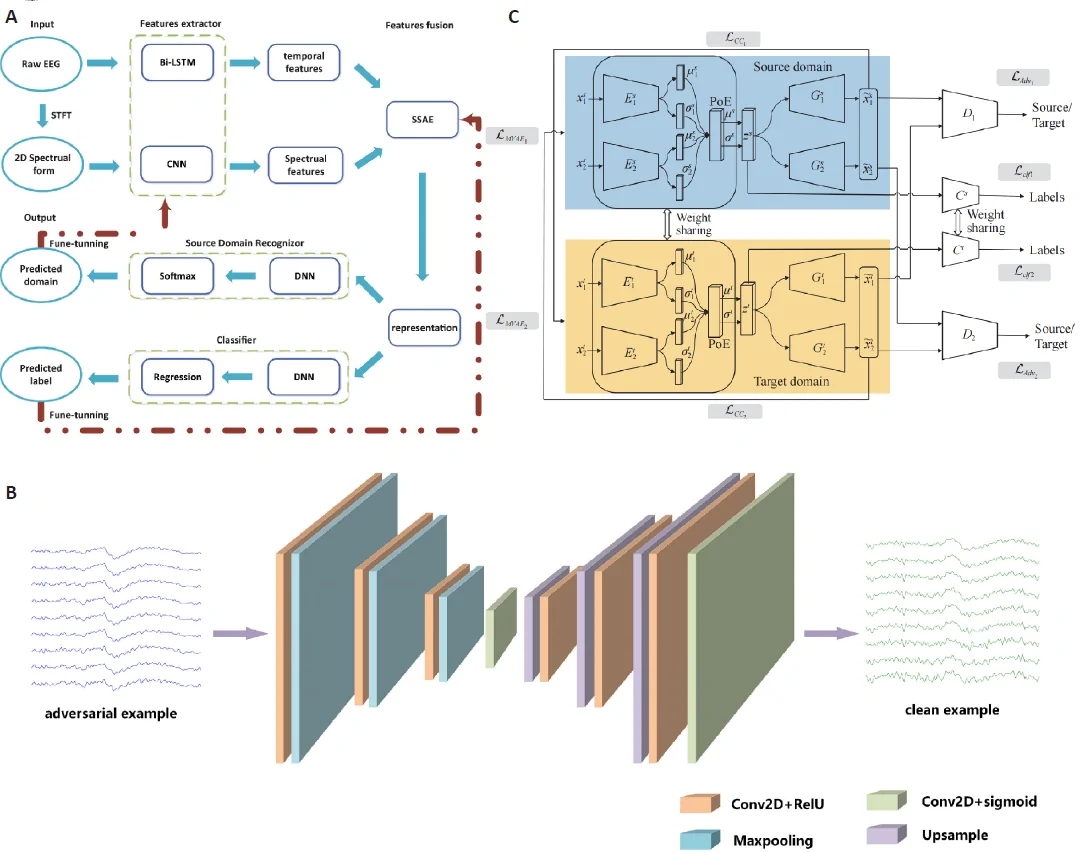
Examples of AE-based decoding algorithms for EEG-BCI. (A) Overview of the MDDL method. Reprinted from Yang et al. (2022). (B) Overview of the DCAE. Reprinted from Ding et al. (2024). (C) Overview of the MMDA-VAE framework. Reprinted from Wang et al. (2022a). 2D: Two-dimensional; AE: autoencoder; BCI: brain‒computer interface; Bi-LSTM: bidirectional long short-term memory; CNN: convolutional neural network; DCAE: denoising convolutional autoencoder; DNN: deep neural network; EEG: electroencephalography; MDDL: multihierarchical discriminative deep learning; MMDA-VAE: multimodal domain adaptive variational autoencoder; SSAE: stacked sparse autoencoder.

A novel multi-hierarchical discriminative deep learning (**Figure 15A**) method based on multi-hierarchical representation fusion was proposed for MI-EEG signals (Yang et al., 2022). The method employed a concurrent framework that integrates bidirectional LSTM and CNN to capture contextual correlations and spectral features of MI-EEG. Additionally, a stacked sparse AE was utilized to fuse these features into a high-level representation, facilitating cross-session and subject training. The experiments on both public and proprietary datasets showed the feasibility and effectiveness of the proposed multi-hierarchical representation fusion model on inter-session (average accuracy: 80.3% for session 1 and 80.6% for session 2 on a public BCI competition IV dataset 2a; 73.4% for session 1 and 74.3% for session 2 on a proprietary dataset constructed by researchers) and inter-subject (71.7% average accuracy) learning. This evidence provides substantial support for the practical implementation of a calibration-free BCI system. A denoising convolutional AE (**Figure 15B**) was presented, which serves as a preprocessing unit preceding the classification model for a defense mechanism against adversarial attacks (Ding et al., 2024). This module effectively neutralized input perturbations while maintaining classifier integrity, thereby demonstrating superior robustness across multiple attack scenarios. Future directions include hybrid defense integration and extension to gray/black-box attack mitigation. A multi-modal domain adaptive variational AE (**Figure 15C**) was presented for EEG-based emotion recognition (Wang et al., 2022a). By combining multi-modal alignment through adversarial learning and cycle-consistency regularization, this framework achieved state-of-the-art performance in low-calibration scenarios across two benchmark datasets, outperforming conventional transfer learning approaches.

#### Deep belief network

DBN is a deep learning model composed of multiple stacked restricted Boltzmann machines. Each restricted Boltzmann machine consists of a visible layer and a hidden layer, with the connection weights between them optimized to minimize the reconstruction error of the data samples (Zuo et al., 2019). The DBN training process employs a bottom-up hierarchical approach, where each layer of the restricted Boltzmann machine independently undergoes unsupervised training to learn the distribution of the data. Through layer-by-layer training and feature propagation, DBN can extract higher-level feature representations, thereby improving the accuracy of tasks such as classification and clustering (Alom et al., 2019).

**Figure 16:**
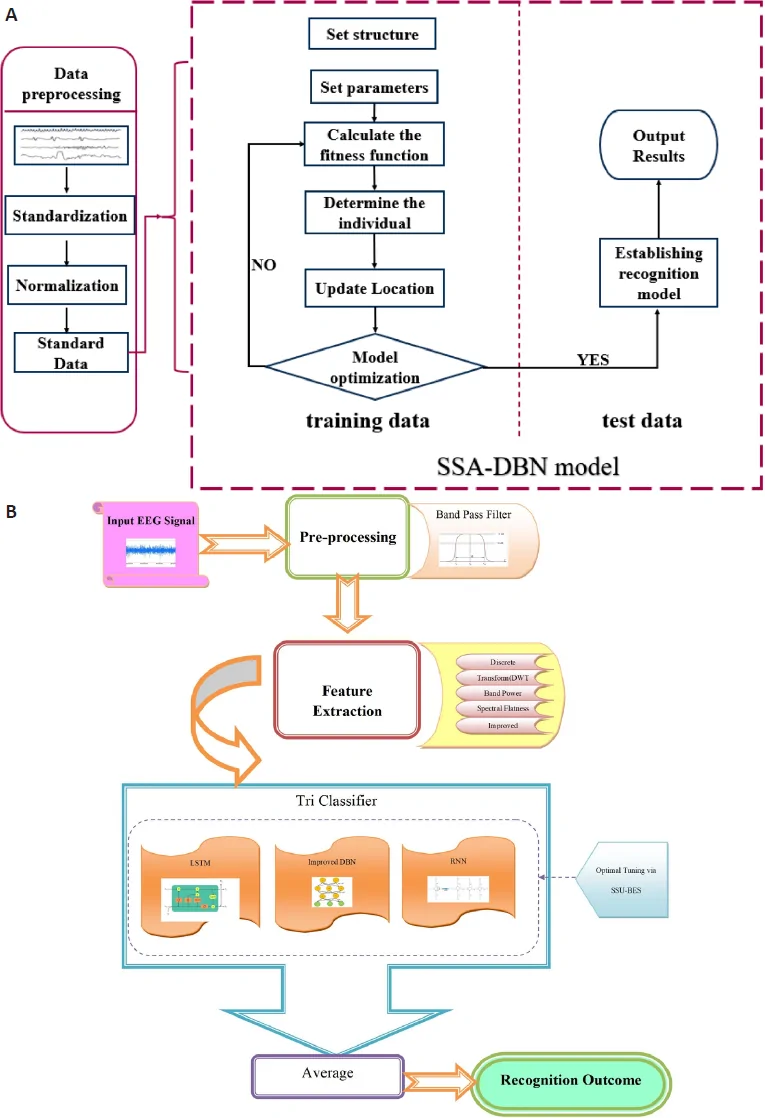
Examples of DBN-based decoding algorithms for EEG-BCI. (A) Overview of the SSA-DBN model. Reprinted from Wang et al. (2023c). (B) Overview of the hybrid optimization-assisted framework for EEG-based emotion recognition. Reprinted from Mohammad et al. (2023). BCI: Brain–computer interface; EEG: electroencephalography; DBN: deep belief network; LSTM: long short-term memory; RNN: recurrent neural network; SSA-DBN: sparrow search algorithm deep belief network.

A sparrow search algorithm-DBN (**Figure 16A**) was proposed to recognize the EEG features extracted by the empirical mode decomposition (Wang et al., 2023c). Experimental validation across three benchmark datasets revealed remarkable classification accuracies of 87.83%, 86.14%, and 87.21% respectively, outperforming conventional methods by significant margins of 10.38%, 9.33%, and 5.57%, respectively. A novel EEG-based emotion recognition model that utilizes a hybrid optimization-assisted framework (**Figure 16B**) was presented (Mohammad et al., 2023). The methodology integrated a tri-model classifier architecture combining LSTM, enhanced DBN, and RNN components whose weights were optimally tuned using a newly proposed model named shark smell updated bald eagle search optimization algorithm. This tri-classifer + shark smell updated bald eagle search demonstrated superior performance (accuracy, F1-score, sensitivity, specificity and precision: 92.19%, 0.9154, 88.40%, 95.66%, and 94.91%, respectively, using valence case; 93.36%, 0.9385, 93.91%, 92.71%, and 93.78%, respectively, using arousal case) in EEG-based emotion recognition compared to existing methods.

#### Transformer

A groundbreaking advancement in artificial intelligence emerged with the development of transformer networks, first introduced in the seminal paper “Attention Is All You Need” (Vaswani et al., 2014). This revolutionary architecture has become a cornerstone in sequential data processing, particularly transforming natural language processing (Alberts et al., 2023; Lund and Wang, 2023). The unique architecture of the transformer, comprising encoder-decoder structures with self-attention mechanisms, enables simultaneous processing of sequential data (e.g., text or time-series) while effectively capturing long-range dependencies between elements. Transformers have emerged as a powerful deep learning architecture for EEG-based BCI decoding, with a multitude of applications explored to date (Pfeffer et al., 2024).

**Figure 17:**
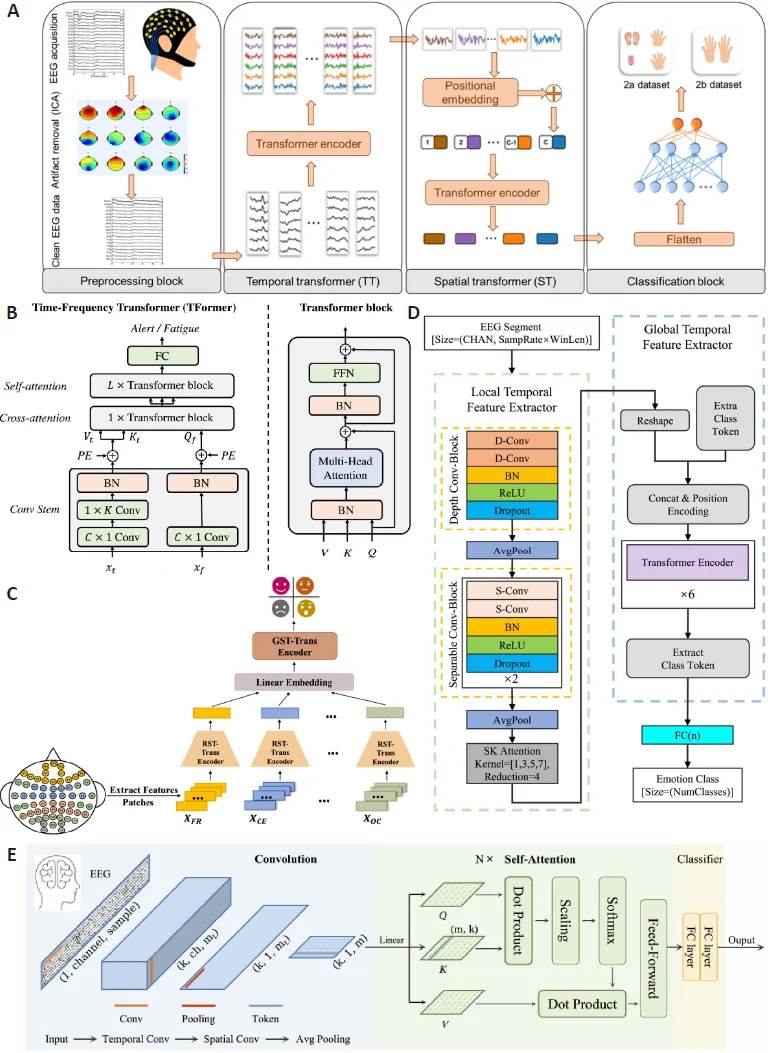
Examples of transformer-based decoding algorithms for EEG-BCI. (A) Overview of the TST model. Reprinted from Hameed et al. (2024). Copyright 2023, Elsevier Ltd. (B) Overview of TFormer. Reprinted from Li et al. (2024c). Copyright 2024, Elsevier Ltd. (C) Overview of R2G-STLT. Reprinted from Cheng et al. (2024). Copyright 2024, Elsevier Ltd. (D) Overview of the MACTN. Reprinted from Si et al. (2024). Copyright 2024, Elsevier Ltd. (E) Overview of the EEG Conformer. Reprinted from Song et al. (2023). BCI: Brain–computer interface; BN: batch norm; Conv: convolutional; D-Conv: depth convolution; EEG: electroencephalography; FC: fully connected; FFN: feedforward network; GST-Trans: global spatial–temporal transformer; MACTN: mixed attention-based convolution and transformer network; RST-Trans: regional spatial–temporal transformer; S-Conv: separable convolution.

Transformers offer distinct advantages in EEG analysis through their capacity for automated feature extraction from raw neural data, significantly reducing reliance on manual feature engineering traditionally required in BCI research. For example, a novel temporal–spatial transformer-based (TST-based) MI classification model, named TST (Hameed et al., 2024; **Figure 17A**), was presented for EEG-BCI using independent component analysis. The temporal transformer component of the TST model is designed to capture the time-domain characteristics of EEG signals and extract relevant features, while the spatial transformer component utilizes an attention mechanism to capture spatial information across channels by addressing inter-channel dependencies. The effectiveness of the TST model is extensively validated on the BCI Competition IV 2a and 2b benchmarks. It outperforms state-of-the-art methods while demonstrating superior accuracy in both subject-dependent and subject-independent experimental settings. Specifically, with subject-dependent methods, it achieved 97.77% on the 2a dataset and 85.90% on the 2b dataset. For subject-independent evaluations, the model yielded 88.72% (2a) and 84.20% (2b) accuracy under five-fold cross-validation, and 93.94% (2a) and 87.29% (2b) accuracy using the leave-one-subject-out protocol.

Transformers use multi-head attention to selectively concentrate on relevant EEG sequence components, effectively mitigating noise interference and enhancing BCI reliability. A time–frequency transformer named TFormer (**Figure 17B**) was proposed for driver fatigue recognition, which can automatically learn global time–frequency patterns from raw EEG data (Li et al., 2024c). TFormer consists of three core components: convolutional stems for input embedding; time–frequency multi-head cross-attention for integrating time-domain patterns into frequency points; and self-attention layers to further model global time–frequency interactions. Experimental results empirically validated the superiority of TFormer over existing methods (average accuracy: 79.68% and 91.19% on SAD and SEEC-VIG datasets, respectively). Notably, this work contributes to the advancement of transformer-based architectures in EEG decoding, and illustrates an innovative approach to leverage transformers for decoding raw EEG data.

The transformer model is adept at managing the sequential characteristics of EEG data, outperforming conventional models (e.g., CNNs and RNNs) in capturing temporal dynamics. Numerous studies have demonstrated its enhanced decoding accuracy. A novel transformer-based hierarchical emotion recognition model termed R2G-STLT (**Figure 17C**) was proposed (Cheng et al., 2024). The regional spatial-temporal transformer encoder was designed to capture electrode-level spatial and contextual data to learn regional spatiotemporal features. Subsequently, the global spatial-temporal transformer encoder extracted global features, showing brain region impacts on emotion recognition. The performance of the model was extensively validated through subject-independent experiments, demonstrating its superiority over several baseline models and state-of-the-art methods (average classification accuracy: 86.22% [arousal] and 80.26% [valence] on Database for Emotion Analysis using Physiological signals [DEAP] dataset, 77.96% on Shanghai Jiao Tong University Emotion EEG Dataset [SEED], and 75.00% on SEED-IV dataset).

Recent innovations focus on hybrid architectures combining transformers with complementary deep learning paradigms. The mixed attention-based convolution and transformer network (**Figure 17D**), a novel hierarchical hybrid model, was proposed for cross-subject EEG emotion recognition (Si et al., 2024). The model employs depth-wise temporal convolution and separable convolution to extract local temporal features, followed by a self-attention-based transformer to integrate sparse global emotional features. Additionally, a channel attention mechanism is incorporated to identify task-relevant channels and capture the relationships between different channels and emotional states. This architecture achieved an 8% improvement in nine-class emotion classification and won the championship of the 2022 World Robot Contest-Emotional BCI. A novel framework named EEG Conformer (**Figure 17E**), which integrates the strengths of both CNNs and transformers, was presented for EEG-based MI and emotion recognition (Song et al., 2023). The model sequentially processed EEG signals through convolutional layers extracting local temporal-spatial features, a self-attention module capturing global correlations, and a fully-connected classifier. Extensive experiments on three public EEG datasets demonstrated the state-of-the-art performance of the EEG Conformer (average accuracy: 78.66%, 84.63%, and 95.30%), showcasing its potential as a new baseline for general EEG decoding.

### Transfer learning

Transfer learning (Pfeffer et al., 2024) is a set of methodologies that leverages knowledge acquired from one specific task (the source domain) to improve model performance on another task (the target domain). By transferring learned patterns or representations, this approach introduces beneficial biases or regularization to address data scarcity in the target domain. Its effectiveness is closely linked to the degree of similarity between the source and target tasks. In EEG-based BCIs, transfer learning scenarios are broadly categorized into three types based on domain variations, namely cross-subject/session, cross-device, and cross-task transfer learning (Wu et al., 2022).

#### Cross-subject/session transfer learning

Cross-subject/session transfer learning utilizes data from multiple subjects or recording sessions (source domains) to optimize calibration for a new subject or session (target domain), typically while maintaining consistent tasks and EEG hardware.

**Figure 18:**
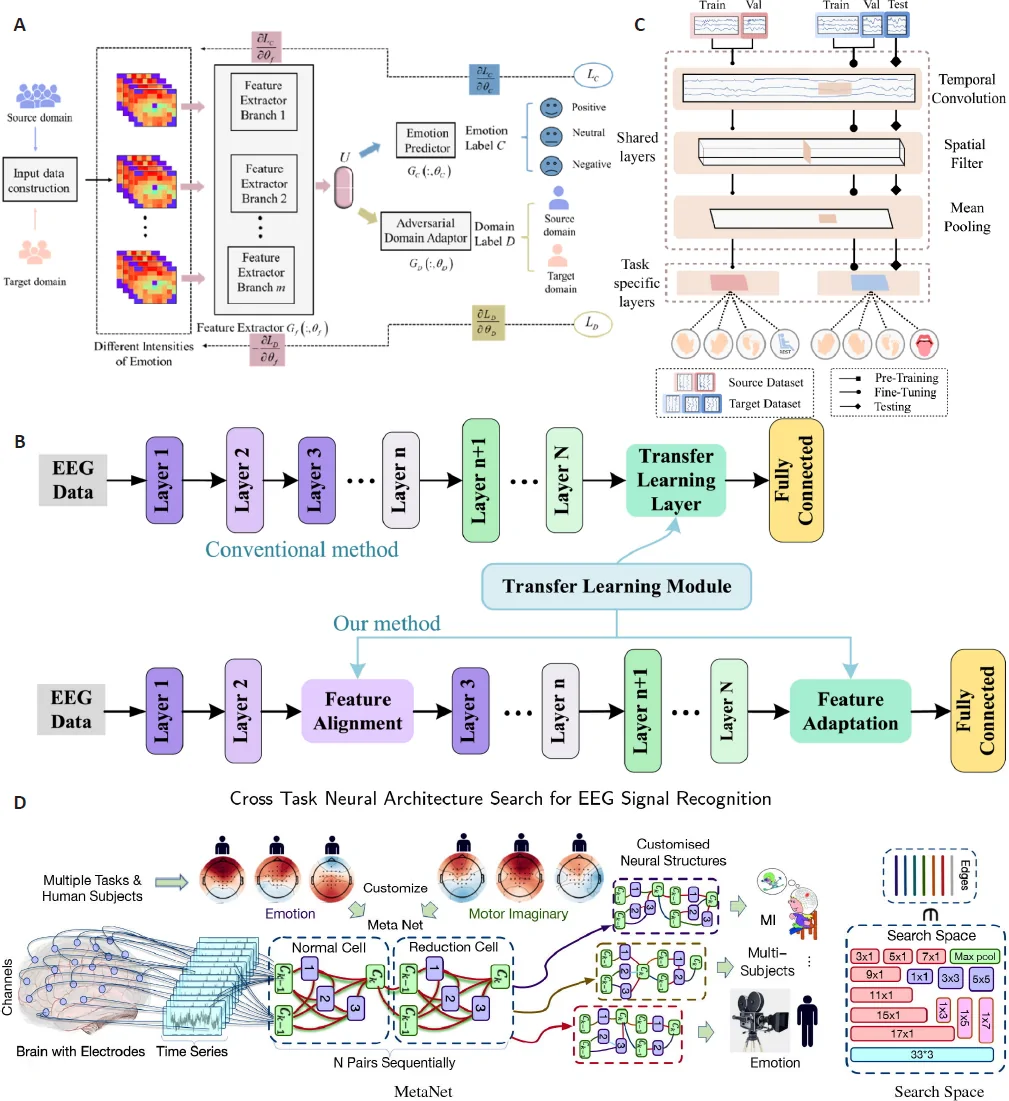
Examples of decoding algorithms using transfer learning for EEG-BCI. (A) Overview of DA-CapsNet. Reprinted from Liu et al. (2024c). Copyright 2023, Elsevier B.V. (B) Overview of the EEGTransferNet. Reprinted from Liang et al. (2024). Copyright 2024 Elsevier Ltd. (C) Overview of the cross-dataset transfer learning method for EEG-based MI signal classification. Reprinted from Xie et al. (2023). Copyright 2023 IOP Publishing Ltd. (D) Overview of CTNAS-EEG. Reprinted from Duan et al. (2023). Copyright 2023, Elsevier B.V. BCI: Brain–computer interface; CTNAS-EEG: cross-task differentiable architecture search for EEG signals; EEG: electroencephalography; MI: motor imagery.

Dual attention-capsule network (DA-CapsNet) (**Figure 18A**), a multi-branch capsule network based on adversarial domain adaption, was proposed for cross-subject EEG-based emotion recognition (Liu et al., 2024c). To precisely capture the spatiotemporal characteristics of emotions in EEG signals, the researchers initially constructed homomorphic difference cube sequences, encoding both electrode positional relationships and temporal dynamics. Additionally, a parallel multi-branch capsule network was developed to extract discriminative features from these sequences and fuse them for emotion classification. This architecture enhances the relevance and richness of emotional representations, while accelerating training efficiency by optimizing feature integration and computational parallelism. Experimental results demonstrated that DA-CapsNet has a rapid processing speed and considerably increases cross-subject emotion recognition accuracy (84.63% on the SEED dataset, 81.63% and 81.09% on valence and arousal, respectively, for the Deep Exposure-Aware Multimodal Content-Based Recommendation [DEAMER] dataset, and 73.62% and 75.25% on valence and arousal, respectively, for the DEAP dataset). Recently, a dynamic interactive network with self-distillation was also proposed for cross-subject EEG-based emotion recognition. It also employed domain adaptation, forming a unified framework to extract subject-invariant multi-modal emotional features (Cheng et al., 2025). On the DEAP dataset, this model achieved similar accuracy for valence and arousal (73.07% and 75.00%, respectively). The EEGTransferNet (**Figure 18B**), an end-to-end modular transfer learning framework, was designed to enhance cross-subject statistical distribution similarity (Liang et al., 2024). Using CNNs as backbone feature extractors, the framework integrated statistical distribution alignment and domain adaptation across network layers to capture both generalizable and domain-specific features. EEGTransferNet demonstrated efficiency and versatility across multiple BCI paradigms, achieving competitive performance compared to existing approaches (area under curve: 74.68% and 68.29% on the MI datasets, 63.17% on the error-related negativity dataset and 65.90% on the rapid serial visual representation [RSVP] dataset).

#### Cross-device transfer learning

Cross-device transfer learning addresses scenarios where knowledge from one EEG acquisition system (source domain) is transferred to optimize calibration for a different device (target domain), with consistent tasks and subjects across devices.

A novel cross-dataset transfer learning method was presented for EEG-based MI signal classification using deep learning (**Figure 18C**; Xie et al., 2023). Inspired by hard parameter sharing in multi-task learning, their approach treats distinct MI datasets as separate tasks within a unified architecture. Four fine-tuning strategies were systematically evaluated, revealing that pre-trained models exhibited superior accuracy, and faster convergence, and enhanced robustness compared to non-pretrained baselines.

#### Cross-task transfer learning

Cross-task transfer learning applies labeled data from analogous or pertinent tasks (source domain), to facilitate the calibration for a novel task (target domain).

Cross-task neural architecture search-EEG (CTNAS-EEG) (**Figure 18D**), a CTNAS framework, was proposed for EEG signal recognition, which can automatically design the network structure across tasks while enhancing recognition accuracy (Duan et al., 2023). Specifically, researchers constructed a cross-task compatible search space and an efficient constrained search method to address EEG signal challenges, and explored for the first time cross-task structural differences via unified search across EEG tasks. Detailed experimental results suggested that CTNAS-EEG could reach state-of-the-art performance on different EEG tasks, achieving accuracies of 77.3%–80.4% on the BCI Competition IV-2a dataset, 76.16/77.01% (specific weights/variable structure) on the SEED-IV dataset, and 74.26/74.21% (specific weights/variable structure) on the SEED-V dataset.

### Conclusion and prospects

**Additional Table 3** summarizes the advantages and disadvantages of EEG-BCI decoding algorithms based on Riemannian geometry, deep learning, and transfer learning.

**Additional Table 3:** The advantages and disadvantages of EEG-BCI decoding algorithms based on Riemannian geometry, deep learning, and transfer learning

Method	Advantage	Disadvantage
Riemannian geometry	• High computational efficiency and simple structure with covariance matrices	• Relies on covariance matrices
	• Robust to noise in moderate-electrode systems	• Insufficient exploration of dynamic signals
	• Dynamic adaptation enhanced by deep learning	• Limited ability to address temporal variability
		• Generalization issues in cross-subject/clinical scenarios
Deep learning	• Automatic feature extraction	• Requires large labeled datasets
	• High accuracy	• Computational cost limits real-time use
	• Architecture diversity	• Sensitivity to electrode placement
	• Long-range dependencies	• Generalization issues across devices
Transfer learning	• Reduced calibration	• Limited cross-task research
	• Device adaptability	• Performance declines with large source-target differences
	• Task scalability	• Lack of unified frameworks
	• Enhanced generalization	• Challenges in cross-device signal alignment

BCI: Brain-computer interface; EEG: electroencephalography.

This section presents a critical review of contemporary EEG-based BCI decoding algorithms, highlighting both methodological advancements and persistent challenges. While remarkable progress has been achieved in BCI research, several fundamental limitations require urgent attention across various approaches.

Covariance matrix-driven Riemannian methods have emerged as a particularly promising framework, gaining substantial traction in the BCI research community. However, four primary limitations constrain their broader application. The exploration of alternative EEG representations beyond conventional covariance matrices, which could enhance robustness, remains insufficient. Existing BCI algorithms require improvements to increase robustness against outliers and to handle the non-stationarity of EEG signals. Moreover, there is requirement for dynamic adaptation strategies to address the temporal variability of EEG.

CNNs have gained popularity in EEG decoding due to their superior pattern recognition capabilities and compatibility with iterative optimization processes. Their hierarchical architecture effectively addresses distribution shifts across experimental paradigms. Nevertheless, critical limitations persist: susceptibility to false positives (Guo et al., 2016; Cubuk et al., 2017); requirement of large datasets prolonging training time (Rakhmatulin et al., 2024); spatial sensitivity to electrode configuration (Rashid et al., 2020); and hyperparameter sensitivity complicating model optimization. These constraints collectively hinder CNN deployment in practical BCI implementations.

LSTM-based EEG-based BCI decoding algorithms have been widely adopted due to their ability to capture long-term dependencies within sequential data. However, they often suffer from parametric complexity, high computational complexity, and limited capability in feature integration (Altaheri et al., 2023). GRU-based methods are structurally simpler than LSTMs and have shown potential for EEG processing efficiency; nevertheless, they remain largely unexplored.

AEs, while effective in EEG decoding, present three key limitations: potential loss of local signal features during encoding (Vallabhaneni et al., 2021); overfitting tendencies with high-dimensional parameter spaces; and high computational complexity restricting real-time applications. Hybrid architectures combining AEs with contemporary deep learning methods show promise for enhanced performance, generalization, and applicability across domains.

DBNs offer unique advantages for small-sample EEG analysis through unsupervised pretraining capabilities (Vallabhaneni et al., 2021). However, practical implementation faces three drawbacks: exponential computational demands with network depth scaling, memory-intensive training processes, and limited cross-subject generalizability due to feature transmission inefficiencies.

Transformer-based approaches demonstrate significant potential through their attention mechanisms but face six development challenges: significant computational demands, large-scale data requirements, real-time processing limitations, cross-domain generalization gaps, interpretability constraints and the carcity of pretrained models (Abibullaev et al., 2023; Pfeffer et al., 2024). Emerging solutions include lightweight variants, multimodal fusion techniques, and optimized temporal processing modules.

Transfer learning has become instrumental in addressing two critical BCI challenges: subject-specific calibration reduction and performance enhancement. However, the majority of research in the domain of transfer learning has concentrated on the transferability of knowledge across different subjects or sessions. While interest in cross-device transfers is increasing, the exploration of cross-task transfers remains largely neglected (Wu et al., 2022). Notably, transfer learning applications extend beyond conventional classifier adaptation to encompass trial alignment optimization (Zanini et al., 2018), adaptive signal filtering (Dai et al., 2018), and transfer-enhanced feature extraction and selection (Chen et al., 2019). Systematic integration of these transfer-enhanced components into unified pipelines could yield substantial performance improvements. The development of comprehensive transfer learning frameworks addressing both device and task heterogeneity represents a crucial frontier for practical BCI deployment.

## Applications

EEG-based BCIs are revolutionizing the way we interact with technology and the world around us. Originally proposed for applications in medicine and neuroscience research, BCIs have expanded into a variety of innovative fields (e.g., education, training, human–computer interaction, and gaming). As the terminal for technical validation, real-world application scenarios use their specific requirements to reversely drive the development of EEG acquisition, paradigm design, and decoding algorithms. These four components form a symbiotic relationship.

### Healthcare

#### Diagnosis

In disease diagnosis, BCIs use EEG signals to detect and diagnose various brain disorders. Recent advancements in artificial intelligence, particularly deep learning algorithms, have significantly enhanced the accuracy of EEG signal analysis for identifying conditions such as AD, PD, and epilepsy, enabling earlier and more precise diagnoses (Shang et al., 2024).

Recent epidemiological data revealed that AD affects approximately 32 million individuals globally with dementia symptoms, while 69 million individuals show early warning signs (prodromal stage) and 315 million individuals exhibit biological markers without clinical symptoms (preclinical phase) (Gustavsson et al., 2023). In this landscape, EEG technology has emerged as an accessible and practical solution for early detection, offering both affordability and portability. Characteristic EEG patterns in patients with AD (i.e., slowed brain waves, disrupted neural coordination, and simplified signal complexity) provide crucial diagnostic clues. Sleep EEG analysis has become particularly valuable for tracking disease progression, though clinicians continue to face challenges in differentiating subtle neurological variations between patients with early-stage disease and healthy seniors (Azami et al., 2023). Advanced predictive deep learning models using EEG readings have shown remarkable success in detecting the crucial transition from mild cognitive impairment to full dementia (Poil et al., 2013; Wang et al., 2024f), while also effectively monitoring disease advancement in patients experiencing accelerated cognitive deterioration (Chetty et al., 2024; Tanaka et al., 2024). Although it is not yet part of standard diagnostic protocols, the ability of EEG to detect concurrent epileptic activity—a significant prognostic factor—reinforces its clinical value in comprehensive patient evaluation (Massot-Tarrús et al., 2022).

The global prevalence of PD surpassed 8.5 million cases in 2019, representing an 81% increase since 2000, with mortality rates doubling to 329,000 deaths during this period (Bhidayasiri et al., 2024). While magnetic resonance imaging remains the gold standard for the diagnosis of PD, EEG has gained attention due to its ability to capture real-time dynamic brain changes linked to disease progression (Stern et al., 1989). Due to their complexity and nonlinearity, EEG signals elude accurate characterization by linear feature extraction methods (Ahamed et al., 2023). The complexity of EEG signals correlates with PD severity, highlighting the importance of nonlinear features in distinguishing healthy from PD EEG signals (Martis et al., 2013). Deep learning methods, such as CNNs, enable automated feature extraction from raw EEG data, circumventing the limitations of manual feature engineering in traditional methods (Oh et al., 2020; Shaban and Amara, 2022).

Epilepsy currently affects 6.38 per 1000 individuals globally, with an annual incidence of 61.44 per 100,000 person-years (Fiest et al., 2017). The unpredictable nature of seizures poses significant physical, psychological, and safety risks to patients (Batista et al., 2024). Significant progress has been made in improving algorithms for preprocessing, feature extraction, and deep learning algorithms. EEG data preprocessing enhancement methods, such as wavelet transform, empirical mode decomposition, and blind source separation, are common but require thresholds. While generative adversarial networks present a sophisticated approach for data augmentation, their application is constrained by implementation complexity and computational costs, whereas window overlapping offers a more straightforward alternative. Additionally, the integration of multiple datasets has been shown to significantly enhance model robustness and generalization capabilities in EEG signal processing applications. Among various feature extraction methods, feature fusion techniques have become popular for comprehensive data analysis; nonetheless, they can lead to redundancy. Selection methods such as Fisher score, group search algorithm, crow search algorithm, and optimal feature selection algorithm can prevent this problem (Subasi et al., 2019; Kapoor et al., 2022). Deep learning combined with manual extraction also enhances performance (Cao et al., 2020). Deep learning algorithms for epilepsy detection and prediction have been utilized in a wide range of approaches. CNN-based architectures remain the most commonly used for seizure detection (Zhang et al., 2024e), while RNNs, generative adversarial networks, and spiking neural networks show growing potential for prediction and real-time applications (Akopyan et al., 2015; Truong et al., 2019). Merging innovations, such as cosine CNNs (Liu et al., 2024a) and capsule networks (Sabour et al., 2017), address inherent CNN limitations in temporal resolution and spatial invariance. Attention mechanisms are increasingly integrated into multimodal frameworks to efficiently manage long-term EEG time-series data.

Despite these achievements, EEG-BCI applications in disease diagnosis are characterized by key challenges. Dataset limitations persist, including small sample sizes that compromise generalizability and risk overfitting, confounding variables such as medication effects on EEG signals, and the impracticality of high-density electrode setups for portable, long-term monitoring (Zhang et al., 2024e). Artificial intelligence model challenges arise from the limited interpretability of deep learning architectures, such as CNNs and RNNs, which undermines clinician trust, alongside issues of standardized reporting and comparability across studies due to heterogeneous datasets and model parameters (Shang et al., 2024). Clinically, real-world deployment of portable, wearable devices with maintained signal quality and computational efficiency for real-time monitoring remains a hurdle, alongside patient compliance issues, such as motion artifacts from restless individuals. In rehabilitation contexts, unaddressed factors (e.g., neurofeedback timing, calibration paradigm engagement, and treatment frequency/intensity) further limit efficacy, while inconsistent study designs impede cross-study comparability and holistic understanding of BCI-based therapeutic impacts. Addressing these challenges—spanning data, model, technical, and clinical domains—is critical to advancing the reliability and utility of EEG-BCI in disease diagnosis and rehabilitation.

#### Rehabilitation

EEG-based BCIs have revolutionized medical rehabilitation for neurodegenerative disorders (e.g., AD, PD), stroke, and mental health conditions (e.g., depression, anxiety, ADHD). These noninvasive systems offer novel pathways to restore communication and motor function in patients with severe impairments (Zhang et al., 2024c).

The progressive cognitive decline of AD patients complicates active BCI engagement. BCIs applied in AD rehabilitation often rely on relatively intact cognitive functions to bypass the peripheral nervous system (Liberati et al., 2012). Galvin-McLaughlin et al. (2022) evaluated EEG neurofeedback via an RSVP keyboard to increase visual attention in mild AD patients. While the results are inconclusive, the protocol provides a foundation for future BCI adaptations in neurodegenerative contexts. For AD patients, cognitive decline impairs their ability to actively engage with EEG-BCI, as tasks such as MI or intentional control rely on relatively intact cognitive functions. Even conditioned responses via EEG-based paradigms show high individual variability and potential weakening over time. Therefore, future research needs to identify a method that bypasses the active participation of patients with BCIs through classical conditioning or other mechanisms (Tayebi et al., 2023).

PD is the second most common neurodegenerative disease, following AD. The application of EEG-based BCI in patients with PD includes adaptive deep brain stimulation (DBS), which adjusts stimulation parameters via local field potential feedback from implanted electrodes. Compared to conventional DBS, adaptive DBS improves motor scores and energy efficiency through optimized stimulation cycles (Pozzi and Isaias, 2022; Smyth et al., 2023). Furthermore, close-loop clinical neural interface has been used in PD rehabilitation. AlphaDBS system, a closed-loop neural interface with artifact-resistant recording and adaptive stimulation capabilities, exemplifies successful clinical translation (Arlotti et al., 2021). Additionally, EEG-based neurofeedback therapy (NFT) enables patients with PD to modulate specific brain rhythms, yielding measurable motor improvements (Buyukturkoglu et al., 2013; Miladinovic et al., 2020). In PD, reduced MI ability, a core component of many EEG-based NFT, impairs feedback control (Linden and Turner, 2016). Existing studies using EEG for tremor detection or prosthetic control have shown potential; however, they are currently in their early stages, with small cohorts, and limited real-world validation (Giuliana et al., 2011). Stroke is the second leading cause of death and the third leading cause of death and disability worldwide. From 1990–2019, there was a substantial increase in the burden of stroke, with a 70.0% increase in incident strokes, a 43.0% increase in deaths from stroke, a 102.0% increase in prevalent strokes, and a 143.0% increase in disability-adjusted life-years lost (Feigin et al., 2022). EEG-based BCIs have been found to primarily enhance neuroplasticity and support patients experiencing motor function impairment, suggesting promising clinical prospects for poststroke patients and those with neurological disorders (Kim et al., 2025). EEG-based BCI-driven interventions promote neuroplasticity and motor recovery through diverse feedback modalities via deep learning technology: visual (Guo et al., 2022; Wang et al., 2024h), robotic (Zhang et al., 2024d), functional electrical (Brunner et al., 2024), VR (Lin et al., 2023a), and multimodal approaches (Wang et al., 2022b). While upper limb rehabilitation dominates research, preliminary evidence supports the efficacy of BCIs in lower limb motor and gait restoration (Blanco-Diaz et al., 2024). To enable broader application of BCI systems in routine rehabilitation practice, further research is needed on the optimal duration, intensity, and frequency of BCI treatment, as well as how to standardize these parameters (Mane et al., 2020). In addition to motor rehabilitation, expanding applications to address cognitive and emotional deficits remains a critical research priority (Yuan et al., 2021).

In 2019, the incidence of depressive disorders increased by 59.3% to 290 million, with an emerging transition in incidence from the young and middle-aged population to the old population (Wu et al., 2024). In the same year, the global burden of disease estimated the global age-standardized incidence and prevalence of ADHD across the lifespan to be 0.061% and 1.13%, respectively, and ADHD accounted for 0.8% of the global mental disorder disability-adjusted life-years (Cortese et al., 2023). BCIs are gaining interest in the realm of mental health, showing promise in NFTs for anxiety, depression, and ADHD (Gonzales et al., 2022; Oganesian and Shanechi, 2024). By adjusting brain activity, these approaches may enhance emotional control and cognitive abilities, paving the way for innovative treatment options.

Emerging evidence highlights several key brain regions as primary targets for DBS in treatment-resistant depression, including the subgenual anterior cingulate cortex, orbitofrontal cortex, nucleus accumbens, ventral capsule/ventral striatum, and bilateral internal capsules (Bergfeld et al., 2016; Kisely et al., 2018; Zhou et al., 2018; Scangos et al., 2021a, b; Zhang et al., 2022). Zhang et al. (2022) demonstrated that bilateral habenular DBS significantly alleviated depressive and anxiety symptoms while enhancing overall health status and quality of life in treatment-resistant depression and bipolar disorder patients. Notably, weaker and more symmetrical oscillatory activities within the habenula were correlated with increased severity of depression and anxiety. Although multiple meta-analyses support the therapeutic efficacy of DBS for treatment-resistant depression (Hurwitz et al., 2022; Reddy et al., 2024), critical limitations persist: existing randomized controlled trials remain scarce and predominantly feature small sample sizes. The current consensus emphasizes the urgent need for large-scale randomized controlled trials to validate these findings and optimize clinical protocols through precise anatomical targeting and parameter modulation (Kisely et al., 2018; Zhou et al., 2018).

ADHD has been linked to atypical patterns of brain wave activity, identifiable through EEG assessments (Lenartowicz and Loo, 2014; Zhang-James et al., 2021). NFT, a neuromodulation approach that trains individuals to consciously regulate their EEG patterns for symptom mitigation, has been extensively studied in pediatric and adolescent populations. Clinical investigations demonstrated sustained improvements in attentional deficits following NFT interventions, though the therapeutic effects on hyperactive and impulsive symptoms exhibited greater variability across studies (Marzbani et al., 2016; Van Doren et al., 2019). Standardized treatment protocols typically target specific EEG biomarkers through established methodologies, including theta/beta ratio modulation, SMR reinforcement, and slow cortical potential regulation (Enriquez-Geppert et al., 2019). A home-based EEG-BCI attention training program has been presented for children with ADHD (Lim et al., 2023). The randomized trial involving 20 participants revealed comparable efficacy between 8-week tablet-administered BCI interventions delivered in domestic versus clinical settings. The home-based protocol demonstrated equivalent therapeutic outcomes while maintaining operational feasibility and safety, thereby presenting a viable treatment alternative that alleviates demands on clinical infrastructure. Despite the therapeutic potential, EEG-BCI has not yet been established as a reliable intervention for psychiatric disorders such as ADHD, primarily due to the nonspecificity of its effects and outcomes (McFarland et al., 2017). While EEG neurofeedback training is emerging as a promising therapeutic approach, rigorous, placebo-controlled, randomized, double-blind trials are necessary. Finally, there is a critical need to directly compare its outcomes with those of mainstream pharmacological and cognitive-behavioral interventions. This would allow researchers to establish comparative metrics that can inform evidence-based clinical decision-making.

### Education

#### Learning state recognition

BCI technology makes a positive contribution to educational learning environments, particularly in learning state recognition. Cognitive and affective BCI systems enable unprecedented insights into neurocognitive processes, facilitating the enhancement of learning strategies and the development of brain-based skill acquisition (Jamil et al., 2021). These technologies establish a data-driven framework for teaching-learning methods, including the measurement of brain workload during learning (Zammouri et al., 2018), the assessment of students’ interest in a particular subject (Moldovan et al., 2017), and attention detection for specific tasks (Zhao et al., 2023). For example, a low-cost EEG platform was proposed to assess students’ attention and estimate academic performance in secondary schools (Fuentes-Martinez et al., 2023). Their innovative platform acquires EEG signals during educational video viewing and subsequent question-answering sessions, calculates the power spectral density of the EEG beta band and correlates it with academic performance, with significant results. This tool offers real-time, objective feedback to improve teaching and learning.

#### Learning style assessment

BCI technology shows promise in psychological research for predicting individual psychological profiles, including depression risk, posttraumatic stress symptoms, and manifestations of the Big Five personality traits (extraversion, neuroticism, conscientiousness, agreeableness, and openness) (Zhang et al., 2021c; Li et al., 2022b). Individual differences in learning strategies are significantly associated with these traits. For example, conscientious and open-minded individuals tend to adopt more effective study habits and achieve greater academic success (Marcela, 2015). Learning styles, which reflect how people naturally absorb and retain information, stem from a mix of cognitive, emotional, and physiological factors that collectively shape how learners perceive and engage with educational material.

Traditional learning style questionnaires now face competition from EEG-based BCI systems, which offer more reliable, objective assessments. For example, an EEG-based learning style recognition mechanism was presented in which a CNN model was used to optimize the accuracy of existing EEG recognition models (Zhang et al., 2021a). It involves labeling learning styles, designing stimuli to evoke internal state differences, collecting EEG data, preprocessing, and training a recognition model. The one-dimensional multiscale CNN model achieved 71.2% accuracy, indicating that brain activity patterns can be used to personalize teaching methods.

#### Learning disability intervention

Approximately 5% of school-aged children experience learning difficulties (Lagae, 2008). These conditions are distinct and persistent across the lifespan, manifesting as various educational challenges at different developmental stages. Their expression is shaped by age, medical background, family history, memory, and intelligence quotient (Alloway, 2009; Locke et al., 2009; Uhre et al., 2024). Facing these challenges, it is important to develop customized diagnostic frameworks and therapeutic interventions to address core deficits and equip learners with adaptive strategies. Recent advances in BCI technology offer novel opportunities for targeted cognitive enhancement training, enabling researchers to address learning disabilities caused by cognitive impairments (Ünlü, 2024).

NFT exemplifies this approach by using BCI systems to monitor neural activity and deliver real-time feedback, thereby capitalizing on the brain’s neuroplasticity to optimize cognitive functioning and behavioral regulation (Marzbani et al., 2016). This nonpharmacological treatment approach has demonstrated therapeutic potential for specific learning disorders, including dyslexia (Taskov and Dushanova, 2022), dysgraphia (Markiewcz, 2017), dyscalculia (Hou et al., 2024), and ADHD (Neuhäußer et al., 2023). By operantly conditioning neural patterns, NFT aims to recalibrate brain dynamics, fostering sustained functional improvements without pharmacological intervention.

#### Challenges and prospects

BCI applications in education remain at a nascent stage, constrained by critical limitations: suboptimal classification accuracy, the absence of standardized protocols (Xu and Zhong, 2018), and insufficient research on real-time identification. Current methodologies overemphasize cognitive process metrics, often neglecting synergistic factors such as affective states and environmental influences that substantially modulate learning efficacy (Jamil et al., 2021). Theoretical integration poses additional challenges, as competing educational frameworks complicate the selection of appropriate models for individualizing BCI interventions. This theoretical plurality risks semantic ambiguity in educational terminology, where identical constructs may reflect divergent conceptual systems (Newton, 2015; Papadatou-Pastou et al., 2021). Furthermore, the validation of BCI efficacy across diverse educational settings remains rare, particularly with respect to longitudinal impacts on metacognitive strategy development and domain-general cognitive enhancement (Andujar and Caprio, 2018). To realize the potential of BCIs in education, future initiatives must prioritize evidence-based pedagogical integration, ensuring alignment with healthy educational practices. Concurrently, commercial advancements in wearable neurotechnology—characterized by wireless connectivity, portability, and user-centric design—are catalyzing the transition from laboratory prototypes to scalable classroom implementations.

### Gaming

BCIs are gaining significant traction in the gaming industry, revolutionizing the way players engage with virtual worlds. These advanced technologies enable direct translation of cognitive states and intentional patterns into in-game actions through neural signal decoding, effectively eliminating dependence on conventional peripherals (e.g., keyboards, mice, joysticks) while enhancing immersion through biocybernetic feedback loops (Sung et al., 2012; Cabañero-Gómez et al., 2018; Cattan et al., 2018; Paszkiel et al., 2021). BCIs redefine the human-game boundary through unprecedented levels of embodied cognition in virtual ecosystems.

Contemporary implementations primarily utilize EEG-BCIs, with particular progress in VR-integrated systems (Bulat et al., 2020). For example, a hybrid SSVEP + P300 BCI system was proposed for controlling avatars in VR gaming, addressing issues such as visual fatigue and low communication rates (Kapgate, 2024). The system was tested via a VR game where the avatar’s movement was controlled by the user’s cortical responses, facilitated by a BCI headset, offering better performance, accuracy, and user comfort. The technological convergence extends beyond VR to encompass extended reality (XR) platforms (Georgiev et al., 2021; Wen et al., 2021), as evidenced by Prapas et al. (2023) in their 3D BCI game called Mind the Move. This Unity-engine implementation leverages Muse 2’s four-channel EEG system with OpenViBE signal processing, achieving 96.94% classification accuracy across three mental states through Riemannian geometry-based feature extraction.

Despite these advancements, critical limitations persist in BCI gaming applications. Approximately 15%–30% of users exhibit BCI illiteracy due to the low quality of the brain signals they produce, which poses an additional barrier to the widespread adoption of BCI systems (Halder et al., 2019; Wen et al., 2021). Current systems require extensive calibration phases (typically 10–20 sessions) to establish reliable decoder models, creating substantial user acclimatization barriers. SNR degradation from extraneous sensory inputs (ambient electromagnetic interference, proprioceptive distractions) remains unresolved. While enhancing cognitive engagement, BCI systems generally demonstrate inferior action‒perception latency compared with traditional inputs (Marshall et al., 2013). Moreover, the decoding algorithm in practical applications is relatively simple and lacks the application of advanced algorithms, such as deep learning.

### Intelligent control and robotics

#### Autonomous driving

One of the most important applications of EEG-based BCI systems is supporting body movement, which is achieved by controlling external prosthetics or driving a vehicle such as a wheelchair or a car. By decoding neural signatures, EEG-BCIs can reveal the cognitive state of drivers, such as fatigue or distraction, thereby enabling real-time safety interventions (Ju and Li, 2024). As a feedback mechanism, BCIs facilitate bidirectional human‒machine interactions, where autonomous systems can learn from human decision-making patterns to refine operational behaviors (Duan et al., 2024). Integrating BCI allows the system to be generalized to new scenarios, learn contextual information and coping strategies from human drivers, and enhance the ability to imitate human behavior (Dong et al., 2024). For example, Mori et al. (2024) proposed a BCI-controlled electric wheelchair using mixed-reality and virtual sound sources, overcoming spatial navigation limitations in conventional assistive devices. The system achieved 70% accuracy in visual stimulus classification and 50% accuracy in auditory recognition, with online testing confirming that target selection capabilities exceed chance levels. The study suggests that performance could be enhanced by improving mixed-reality technology or using more discernible sounds.

Despite these advancements, challenges in technology, the user experience, system responsiveness, safety and ethics, and practical application barriers must be addressed. Technical limitations include signal sensitivity to noise, low spatial resolution, and inconsistent device quality. While comfort assessment has been extensively studied in autonomous driving research, comprehensive models integrating primary comfort factors and their interdependencies remain conspicuously absent (Domova et al., 2022). System performance is further compromised by persistent delays and instability that undermine real-time interaction capabilities. Safety and ethical issues, such as vulnerable user protection, neuroprivacy concerns, and accountability frameworks, also require attention. In practical applications, BCI systems may encounter uncharacterized environmental interference, motion artifacts, and ecological validity gaps, limiting the simulation of real-world complexity. Addressing these challenges requires coordinated progress in three areas: signal acquisition hardware improvements, human-centered interface design optimization, and the establishment of ethical guidelines for neuroadaptive systems. Future research should prioritize ecological validation studies and hybrid architectures that combine BCIs with other biosignal modalities.

#### Exoskeleton

Driven by progress in sensor technology, wearable robotics, computational power, and innovations such as 3D printing and BCIs, exoskeletons are rapidly evolving into highly capable assistive devices. These wearable mechanical systems augment human physical performance and are increasingly deployed across medical rehabilitation, military operations, and industrial labor support. Modern designs now encompass full-body exoskeletons, as well as specialized upper- and lower-limb variants, providing targeted assistance to joints such as the shoulders, elbows, wrists, and ankles (Ferris et al., 2005; Al-Quraishi et al., 2018; Tariq et al., 2018).

EEG-BCI-controlled upper-limb exoskeletons are primarily used for the rehabilitation of diseases such as stroke (Tang et al., 2024) and disability assistance (Crea et al., 2018). For example, a novel upper-limb rehabilitation exoskeleton system called VR-ULE was presented for stroke rehabilitation, which combines MI-BCI with VR (Tang et al., 2023b). The immersive VR environment enhances neuroplasticity and addresses the limitations of conventional MI-BCIs. Offline experiments demonstrated a significantly higher MI classification accuracy with VR cues (86.72% ± 2.99%) than with non-VR cues. During online trials, the system achieved an 88.48% ± 5.84% average task completion rate, underscoring its efficacy in supporting poststroke recovery. This system is expected to be extended to multijoint rehabilitation for other diseases, such as spinal cord injury (SCI). Recently, a novel hand rehabilitation exoskeleton system based on the MI paradigm was developed for stroke patients with hand motor impairments (Tang et al., 2025). This system innovatively integrates EEGNet (Lawhern et al., 2018), a general EEG classification network based on a CNN, with graph convolutional networks to extract spatiotemporal and topological features effectively. An offline classification accuracy of 89.84% was achieved in the initial evaluations, whereas the online training experiment recorded a task completion rate of 87.75%, highlighting its potential for practical clinical applications in motor function rehabilitation.

Lower-limb EEG-BCI exoskeletons are utilized for diverse applications, including SCI rehabilitation (Capogrosso et al., 2016), poststroke gait training (Frolov et al., 2017), mobility assistance for bedridden patients (He et al., 2018), and operation in extreme environments (Gancet et al., 2011). For example, VR-enhanced MI was integrated with a BCI to control a lower-limb exoskeleton for rehabilitation, which may apply to patients who have suffered a stroke, SCI, hemiplegia, or paraplegia (Lin and Sie, 2024). By implementing a cumulative distribution function autoleveling method to optimize signal processing, their approach achieved 75.35% accuracy in open-loop testing and 74% accuracy in closed-loop rehabilitation trials, validating the feasibility of VR-enhanced BCI systems for lower-limb motor recovery. Another research team also developed a VR-enhanced MI-BCI for controlling a lower-limb exoskeleton, which was evaluated in two patients with SCI and 10 healthy participants (Ferrero et al., 2023). For patients with SCI, the system demonstrated high true positive ratios (77.71% and 76.67% for the static model and 69.17% and 65.5% for the motion model).

The continuous optimization of feature extraction and classification algorithms for EEG signals has significantly enhanced the control accuracy and real-time performance of EEG-BCI exoskeletons, enabling more precise alignment with user intent and smoother motion execution. As application scenarios expand—from clinical rehabilitation to industrial and military use—the integration of BCI exoskeletons with complementary technologies (e.g., artificial intelligence, haptic feedback) is fostering richer interactive experiences and multifunctional training protocols (Yousefi-Koma et al., 2023; Ma et al., 2024). Nevertheless, challenges persist, including device miniaturization, seamless multimodal data integration, adaptive control strategies, and rigorous clinical validation to ensure safety and efficacy (Kim et al., 2022; Mathias et al., 2025). Addressing these hurdles will be critical to unlocking the full potential of EEG-BCI exoskeletons in real-world settings.

#### Space exploration

EEG-based BCIs have been increasingly used in the field of space exploration. By facilitating direct neural interaction between astronauts and spacecraft systems, this innovation enables more efficient command transmission in confined or extreme extraterrestrial environments, thereby minimizing delays and potential errors inherent in manual operations (Rossini et al., 2009). Beyond spacecraft operations, this technology empowers astronauts to remotely manipulate robotic assistants and exploratory probes, performing extravehicular activities and maintenance tasks without direct physical intervention—significantly reducing astronauts’ exposure to hazardous environments during critical procedures (de Negueruela et al., 2011). The system’s capacity for real-time neurocognitive monitoring further enhances mission safety, as it continuously tracks brain activity patterns to detect early signs of fatigue, stress, or attentional decline. This capability not only helps mitigate operational risks but also provides actionable insights to optimize crew performance and well-being during prolonged missions (Hummadi and Chatterjee, 2021). As space exploration continues to push the boundaries of human capabilities, the integration of BCI technology holds significant promise for advancing our understanding of the universe and expanding our presence beyond Earth.

### Clinical trial

The ChiCTR is the national clinical trial registration center designated by the Ministry of Health of China to represent China in the World Health Organization International Clinical Trials Registry Platform. It is a non-profit academic institution. According to ChiCTR data (**Additional Table 4**), among the 64 clinical trials on EEG-based BCI, stroke is the most common target disease, accounting for 67.2% of cases. Subjective cognitive impairment is the second most common target disease (10.9%), followed by cognitive dysfunction (4.7%). Of the 64 studies, three are non-rehabilitation studies. Among the remaining 61 rehabilitation studies, 55 focus on motor function rehabilitation, and 12 on cognitive function rehabilitation (with possible overlap between studies). The earliest study was initiated in 2012, and the number of new studies added each year has generally increased, peaking in 2024 with 23 studies (35.9% of all studies). Among all study types, interventional studies constitute the vast majority (93.8%). According to the clinical trial phases regulated by the National Medical Products Administration, 40 studies (62.5%) are in Phase 0, six studies (9.4%) are in Phase 1, and eight studies (12.5%) are categorized as new treatment measure clinical studies. It was not possible to assign a phase to 10 studies (15.6%).

**Additional Table 4:** Clinical trials of EEG-based BCIs in the Chinese Clinical Trial Registry

		Count	Percentage
**Disease**			
Stroke		43	67.2
Spinal cord injury		7	10.9
Cognitive dysfunction		3	4.7
Cerebral palsy		2	3.1
Other		2	3.1
Left cerebral hemisphere damage		1	1.6
Motor neuron disease		1	1.6
Disorder of consciousness		1	1.6
Depressive disorder		1	1.6
Brain injury		1	1.6
Amyotrophic lateral sclerosis		1	1.6
High cervical spinal cord tetraplegia		1	1.6
**Target rehabilitation function (overlapping in different studies)**			
Movement	Upper limb	23	-
	Movement	18	-
	Lower limb	12	-
	Swallowing	2	-
Cognition		12	-
Brain plasticity		5	-
Nonrehabilitation research		3	-
**Start date**			
2025		1	1.6
2024		23	35.9
2023		9	14.1
2022		11	17.2
2021		7	10.9
2020		8	12.5
2019		1	1.6
2018		1	1.6
2017		1	1.6
2013		1	1.6
2012		1	1.6
**Study type**			
Interventional study		60	93.8
Observational study		2	3.1
Diagnostic trial		1	1.6
Etiology/related factors research		1	1.6
**Phase**			
Phase 0		40	62.5
Phase 1		6	9.4
Not applicable		10	15.6
New treatment measure clinical study		8	12.5

BCI: Brain-computer interface; EEG: electroencephalography.

ClinicalTrials.gov is a clinical trial database jointly developed by the National Library of Medicine and the U.S. Food and Drug Administration (FDA). It provides information regarding clinical research studies to the public, researchers, and healthcare professionals. We retrieved 174 clinical studies on EEG-based BCI from ClinicalTrials.gov (**Additional Table 5**). The top 10 conditions/diseases in these studies are: stroke; SCI; amyotrophic lateral sclerosis; tetraplegia; healthy; hemiparesis; locked-in syndrome; muscular dystrophies; mild cognitive impairment; and motor neuron disease (with possible overlap between studies). Consistent with ChiCTR data, stroke and SCI rank first and second, respectively. Among the 174 studies, 70 have treatment as their primary purpose (40.2%). However, 12.1% did not disclose their purpose, and 11.5% disclosed “other” as their purpose. Studies with the purpose of basic science and device feasibility also account for a relatively high proportion, at 11.5% and 9.2%, respectively. The earliest study was initiated in 2005, 7 years earlier than the ChiCTR data. The number of new studies added each year generally increased, peaking in 2022 with 18 studies (10.3% of all studies), five fewer than the highest number shown in the ChiCTR data (23 in 2024). Among all study types, interventional studies are the most common, accounting for 87.9% of all investigations. Currently, the status of all studies registered in ClinicalTrials.gov is as follows: not yet recruiting (*n* = 12, 6.9%); recruiting (*n* = 37, 21.3%); enrolling by invitation (*n* = 2, 1.1%); active, not recruiting (*n* = 4, 2.3%); and completed (*n* = 76, 43.7%). However, 27 studies (15.5%) have an unknown status, and the last known status of these studies was as follows: recruiting; not yet recruiting; or active, not recruiting but has passed its completion date, and the status has not been verified within the past 2 years. Twelve studies (6.9%) were discontinued generally due to high mortality risk or serious adverse event risk, or because the therapy was proven ineffective and continuing therapy would not be in the best interest of the participants. Four studies (2.3%) had withdrawn informed consent. According to the U.S. FDA definitions, the studies are in Phase 0 (*n* = 3, 1.7%), Phase 1 (*n* = 3, 1.7%), Phase 1|Phase 2 (*n* = 2, 1.1%), Phase 2 (*n* = 3, 1.7%), Phase 2|Phase 3 (*n* = 1, 0.6%), and Phase 4 (*n* = 1, 0.6%). It was not possible to assign a phase to 140 studies (80.5%), indicating that the trials were conducted without U.S. FDA-defined phases, including trials of devices or behavioral interventions.

**Additional Table 5:** Clinical trials on EEG-based BCIs at ClinicalTrials.gov

	Count	Percentage
**Top 10 conditions/diseases (overlapping in different studies)**		
Stroke	63	-
Spinal cord injury	23	-
Amyotrophic lateral sclerosis	22	-
Tetraplegia	13	-
Healthy	9	-
Hemiparesis	7	-
Locked-in Syndrome	7	-
Muscular dystrophies	7	-
Mild cognitive impairment	6	-
Motor neuron disease	6	-
**Primary purpose**		
Treatment	70	40.2
Unknown	21	12.1
Other	20	11.5
Basic science	20	11.5
Device feasibility	16	9.2
Supportive care	13	7.5
Health services research	6	3.4
Prevention	4	2.3
Diagnostic	4	2.3
**Start date**		
2025	4	2.3
2024	16	9.2
2023	14	8
2022	18	10.3
2021	12	6.9
2020	9	5.2
2019	14	8
2018	13	7.5
2017	10	5.7
2016	6	3.4
2015	12	6.9
2014	9	5.2
2013	12	6.9
2012	4	2.3
2011	8	4.6
2010	6	3.4
2009	1	0.6
2008	3	1.7
2007	2	1.1
2005	1	0.6
**Study type**		
Interventional study	153	87.9
Observational study	21	12.1
**Study status**		
Not yet recruiting	12	6.9
Recruiting	37	21.3
Enrolling by invitation	2	1.1
Active, not recruiting	4	2.3
Completed	76	43.7
Terminated	12	6.9
Withdrawn	4	2.3
Unknown	27	15.5
**Phase**		
Not applicable	140	80.5
Early Phase 1 (Phase 0)	3	1.7
Phase 1	3	1.7
Phase 1|Phase 2	2	1.1
Phase 2	3	1.7
Phase 2|Phase 3	1	0.6
Phase 4	1	0.6
Unknown	21	12.1

BCI: Brain-computer interface; EEG: electroencephalography.

In China, EEG-based BCI clinical trials are primarily focused on the field of rehabilitation, especially research on stroke and spinal cord injuries. Globally, the number of EEG-based BCI clinical trials is higher, and the range of diseases studied is broader, including stroke and spinal cord injuries, as well as amyotrophic lateral sclerosis, quadriplegia, and locked-in syndrome. In recent years, the number of related studies in China has shown an increasing trend, reaching a peak in 2024. Compared to China, EEG-based BCI clinical trials were initiated earlier globally and peaked in 2022; nevertheless, the growth rate in recent years has been slower than that observed in China. Stroke is the most common target disease in EEG-based BCI research, indicating its significance and urgency for clinical applications. Motor function rehabilitation is the main research direction, particularly the recovery of upper limb motor function, which may be related to the high demand from patients with stroke and spinal cord injuries. Research on cognitive function rehabilitation is relatively less. However, with the increasing number of patients with cognitive impairments, it may become a research hotspot in the future. Interventional studies dominate in both ChiCTR or ClinicalTrials.gov, indicating that EEG-based BCI technology is moving from the laboratory to clinical applications. Studies in ChiCTR are mainly in Phase 0, while a significant proportion of studies in ClinicalTrials.gov are in an undefined stage, which may reflect differences in the definition of clinical trial stages across countries and regions. Some studies in ClinicalTrials.gov have been terminated due to high mortality risks or serious adverse events, suggesting that EEG-based BCI technology continues to face challenges in terms of safety and efficacy in clinical applications.

## Limitations

While efforts were made to comprehensively survey the EEG-BCI literature, some recent or niche studies might have been overlooked due to the rapidly evolving nature of the field and potential restrictions in database coverage. Additionally, although clinical trials were included, the review does not analyze the practical clinical implementation of EEG-BCI systems. Lastly, most discussed studies rely on small sample sizes or controlled laboratory settings, raising questions regarding the generalizability of the reported findings to diverse populations and their ecological validity. These limitations highlight opportunities for more systematic reviews with standardized criteria and broader interdisciplinary perspectives in this dynamic research domain.

## Conclusions and Prospects

**Figure 19:**
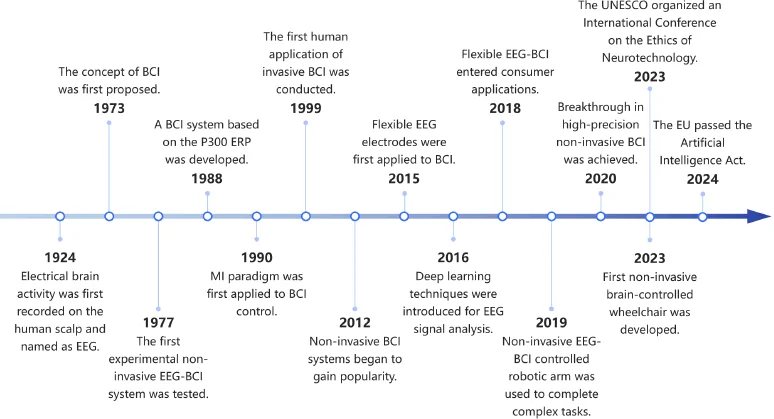
Timeline of the development of EEG-based BCIs. Since the first recording of EEGs in 1924, BCIs have undergone rapid development, from continuous innovations in invasive and noninvasive technical approaches to the current era of widespread adoption of consumer devices, key breakthroughs in handling complex tasks, technological innovations in high-precision control, in-depth deliberation on ethical issues and the gradual improvement of regulatory frameworks. The entire field is experiencing multidimensional epoch-making leaps. BCI: Brain–computer interface; EEG: electroencephalography; EU: European Union; UNESCO: United Nations Educational, Scientific and Cultural Organization.

EEG-based BCI technologies have made significant strides in recent years, offering promising applications across various domains such as healthcare, education, and intelligent control. The advancements in EEG acquisition methods, BCI paradigms, and decoding algorithms have enhanced the accuracy and usability of these systems (**Figure 19**).

However, the practical implementation of EEG-BCI remains constrained by multifaceted challenges spanning interdisciplinary domains. Primarily, EEG signals exhibit inherent limitations due to their biophysical characteristics; specifically, their microvolt-level amplitude results in a low SNR, rendering them susceptible to contamination from physiological artifacts (e.g., ocular movements, electromyographic activity) and environmental interference. Furthermore, the spatial resolution of scalp-recorded EEG is inherently restricted by signal attenuation through cranial tissues, impeding precise localization of cortical neural dynamics. Cross-subject variability in electrophysiological patterns and intra-subject signal non-stationarity caused by fluctuating physiological states (e.g., fatigue, attentional shifts) further undermine the cross-participant generalizability of decoding algorithms. At the hardware level, a trade-off persists between user-friendliness, portability, and signal fidelity in noninvasive EEG systems. Conventional wet electrodes necessitate conductive gel application and prolonged setup times, whereas dry electrodes, despite improved usability, remain vulnerable to motion artifacts and contact impedance variations. Semi-dry electrodes, with flexible material-enabled automatic electrolyte management, demonstrate promising potential. Nevertheless, they are characterized by technical challenges including user-dependent maintenance in earlier designs, insufficient signal quality validation, unaddressed circuit integration requirements, and the need for advancements in wireless recording, machine learning, and real-time processing for practical implementation. Real-time signal acquisition and processing delays in dynamic environments additionally compromise the responsiveness of closed-loop BCI systems.

BCI usability is hindered by prolonged user training requirements. MI paradigms often necessitate weeks to months of calibration for proficiency. Modality-specific fatigue further limits accessibility. SSVEP systems induce visual fatigue from flicker stimuli, excluding visually impaired users, while P300 paradigms demand sustained attention, exacerbating cognitive fatigue. Hybrid BCI paradigms, despite improved accuracy, impose heightened cognitive loads through multimodal feedback.

From a computational perspective, the non-stationary and nonlinear nature of EEG signals imposes rigorous demands on feature engineering and decoding methodologies. While time-frequency decomposition and spatial filtering techniques are widely employed, their efficacy remains task-dependent and subject to individual variability. Deep learning approaches, though promising for automated feature extraction, face constraints due to the labor-intensive annotation and limited scale of EEG datasets. Moreover, model generalization across sessions or participants often necessitates adaptive calibration or few-shot learning frameworks.

In practical applications, EEG-BCI systems confront challenges in environmental adaptability, particularly in mitigating motion artifacts during mobile use, while high user training costs and steep learning curves hinder widespread adoption. The inherent information constraints of unimodal EEG have spurred interest in hybrid BCI architectures integrating complementary modalities (e.g., fNIRS, eye-tracking) and advanced decoding algorithm (e.g., deep learning, transfer learning), though increased system complexity introduces computational integration bottlenecks. Ethical and safety considerations further complicate deployment, encompassing risks of neural data privacy breaches, ethical debates surrounding neuroenhancement, and stringent regulatory requirements for medical-grade device robustness.

While the proliferation of clinical trials investigating EEG-based BCI technologies reflects growing recognition of their therapeutic potential, several critical limitations impede robust clinical translation. Methodological heterogeneity across studies—manifested through inconsistent outcome measures, small sample sizes (typically < 30 participants), and variable experimental protocols—compromises cross-trial comparability and evidence hierarchy. Persistent challenges in signal reliability (e.g., inter-session variability, environmental interference) and decoding accuracy under real-world conditions raise concerns regarding clinical safety profiles. This is evidenced by trial discontinuations attributed to unpredictable system performance. Furthermore, the expanding scope of BCI applications spanning multi-disease paradigms (e.g., stroke, amyotrophic lateral sclerosis, SCI) and heterogeneous outcome measures necessitates standardized efficacy evaluation frameworks, particularly regarding long-term neuroplasticity assessments. Regulatory ambiguity persists as current guidelines struggle to address the unique technical risks of adaptive neural interfaces, including neurofeedback-induced plasticity and device-brain interaction dynamics. These limitations underscore the imperative for large-scale multicenter trials, harmonized reporting standards, and mechanistic studies elucidating neurophysiological correlates of clinical outcomes.

The key improvement directions for EEG-BCI, summarized in **Additional Table 6**, underscore the need for integrated progress across multiple domains. Future advancements necessitate synergistic innovations in biosensing technologies (e.g., flexible electrodes, biocompatible implants) and adaptive computational frameworks. Enhancing cross-user generalizability through transfer learning and meta-learning paradigms, alongside the development of multimodal fusion strategies and energy-efficient embedded systems, represents a critical pathway forward. Concurrently, establishing standardized data protocols, open-source toolkits, and ethical guidelines will accelerate the translation of EEG-BCI technologies from laboratory prototypes to clinical rehabilitation, neuromodulation, and human-computer interaction applications. Addressing these challenges demands sustained interdisciplinary collaboration across neuroscience, materials science, and artificial intelligence, balancing technical feasibility with societal acceptance to unlock the transformative potential of EEG-BCI systems.

**Additional Table 6:** Summary of future directions for EEG-based BCIs

Main directions	Detailed description
Biosensing technology	Flexible and biocompatible electrodes balancing signal quality, user comfort, and portability Wireless, real-time, and miniaturized systems High-spatial-resolution techniques combining other neuroimaging modalities
Generalizable algorithm	Deep learning algorithms balancing complexity, performance, and interpretability for multicenter, large sample multisource longitudinal datasets Comprehensive transfer learning frameworks addressing subject, session, device, and task heterogeneity Multimodal and hybrid algorithms bringing multiple acquisition equipment, hybrid paradigms, and complex applications
Usability and accessibility	User-centric design with little training time, user-friendly systems, and fast response speeds Inclusive paradigms integrating multiple physiological signals, featuring fatigue resistance and low cognitive load
Clinical translation	Harmonized protocols Standardized outcome measures Open-source toolkits Regulatory Frameworks for ethical guidelines, security guarantee, and responsibility assignment

BCI: Brain-computer interface; EEG: electroencephalography.

## Additional files:

***Additional Table 1:*** *The advantages and disadvantages of noninvasive, invasive and minimally invasive EEG electrodes.*

***Additional Table 2:*** *The advantages and disadvantages of the MI, SSVEP, P300, and hybrid paradigms*

***Additional Table 3:*** *The advantages and disadvantages of EEG-BCI decoding algorithms based on Riemannian geometry, deep learning, and transfer learning.*

***Additional Table 4:*** *Clinical trials of EEG-based BCIs in the Chinese Clinical Trial Registry*

***Additional Table 5:*** *Clinical trials on EEG-based BCIs at ClinicalTrials.gov*

***Additional Table 6:*** *Summary of future directions for EEG-based BCIs.*

## Data Availability

*All relevant data are within the paper and its Additional files.*
